# NanoSIMS combined with fluorescence microscopy as a tool for subcellular imaging of isotopically labeled platinum-based anticancer drugs†

**DOI:** 10.1039/c3sc53426j

**Published:** 2014-06-06

**Authors:** Anton A. Legin, Arno Schintlmeister, Michael A. Jakupec, Mathea S. Galanski, Irene Lichtscheidl, Michael Wagner, Bernhard K. Keppler

**Affiliations:** Institute of Inorganic Chemistry, University of Vienna Waehringer Str. 42 A-1090 Vienna Austria bernhard.keppler@univie.ac.at; Research Platform “Translational Cancer Therapy Research”, University of Vienna Waehringer Str. 42 A-1090 Vienna Austria; Large-Instrument Facility for Advanced Isotope Research, University of Vienna Althanstrasse 14 A-1090 Vienna Austria; Department of Microbiology and Ecosystem Research, Division of Microbial Ecology, University of Vienna Althanstrasse 14 A-1090 Vienna Austria; Core Facility of Cell Imaging and Ultrastructure Research, University of Vienna Althanstrasse 14 A-1090 Vienna Austria

## Abstract

Multi-elemental, isotope selective nano-scale secondary ion mass spectrometry (NanoSIMS) combined with confocal laser-scanning microscopy was used to characterize the subcellular distribution of ^15^N-labeled cisplatin in human colon cancer cells. These analyses indicated predominant cisplatin colocalisation with sulfur-rich structures in both the nucleus and cytoplasm. Furthermore, colocalisation of platinum with phosphorus-rich chromatin regions was observed, which is consistent with its binding affinity to DNA as the generally accepted crucial target of the drug. Application of ^15^N-labeled cisplatin and subsequent measurement of the nitrogen isotopic composition and determination of the relative intensities of platinum and nitrogen associated secondary ion signals in different cellular compartments with NanoSIMS suggested partial dissociation of Pt–N bonds during the accumulation process, in particular within nucleoli at elevated cisplatin concentrations. This finding raises the question as to whether the observed intracellular dissociation of the drug has implications for the mechanism of action of cisplatin. Within the cytoplasm, platinum mainly accumulated in acidic organelles, as demonstrated by a direct combination of specific fluorescent staining, confocal laser scanning microscopy and NanoSIMS. Different processing of platinum drugs in acidic organelles might be relevant for their detoxification, as well as for their mode of action.

## Abstract 2

NanoSIMS combined with fluorescence microscopy reveals subcellular structures in cancer cells where ^15^N-labeled cisplatin is accumulated, with implications for Pt–N bond integrity.

## Graphical abstract

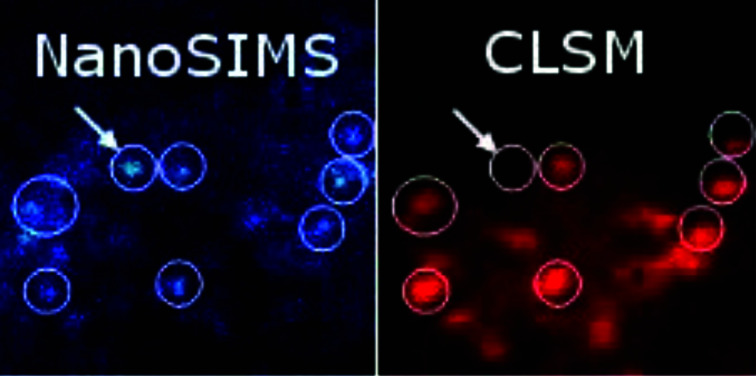

## Introduction

Platinum drugs play an important role in anticancer chemotherapy, predominantly due to their high curative potential in certain malignancies. In the past decades, the development of novel antitumour metal compounds was based mainly on experience gained from investigation of platinum drugs.^1–3^ Whilst the limited spectrum of platinum-responsive tumours, severe side-effects and resistance induction prompted the development of metal-based complexes with central atoms other than platinum, cisplatin (*cis*-PtCl_2_[NH_3_]_2_, CDDP) is still the most widely applied metal-based anticancer agent, showing pronounced efficacy most notably in testicular, head-and-neck and ovarian cancer treatment.

CDDP is generally believed to exert its antitumour effects by DNA adduct formation, thereby disturbing DNA functionality.^4,5^ However, only 1–10% of cisplatin accumulated in the cell is supposed to reach the nuclear DNA.^2,6^ In this context, it is interesting to note that cisplatin can also induce apoptosis in the absence of nuclear DNA, through caspase activation in HCT-116 colon cancer cytoplasts and in enucleated mouse kidney proximal tubule cells.^6,7^

Notwithstanding its high curative potential, cisplatin treatment is associated with undesirable side effects. Major adverse effects are neurotoxicity, ototoxicity and nephrotoxicity, which were shown to be highly dependent on the action of reactive oxygen and nitrogen species.^8,9^ Cisplatin interactions with mitochondrial and lysosomal functions were reported to be involved in peripheral neuropathy.^10^ Although the interaction of cisplatin with tumour cells has been investigated in great detail, the mechanisms of its selectivity, resistance, and toxicity are not completely understood.

Generally, visualisation of the subcellular distribution of pharmaceutical agents can yield valuable information on the location of the sites relevant for biological activity, as well as for detoxification. The subcellular distribution of platinum-based drugs can be studied by carrying out cellular fractionation followed by spectrometric analysis, such as atomic absorption spectroscopy (AAS) or inductively coupled plasma mass spectrometry (ICPMS), which allows an estimation of platinum accumulation in certain compartments of the cell.^11–13^ Due to the risk of cross-contamination of fractions and partial loss of the analyte during sample preparation, there is still an urgent need for methods which are applicable to intact single cells. Attempts have been made to study the subcellular distribution of fluorescently-labeled platinum complexes by fluorescence microscopy.^14–17^ However, fluorescent moieties may affect the (sub)cellular transport, or may be cleaved off by the metabolic activity of the cell. Amongst the element specific imaging techniques, synchrotron based X-ray fluorescence microscopy shows great potential for highly sensitive, quantitative and oxidation state specific mapping of elements with *Z* > 11.^18,19^ X-ray beam focusing optics and high-sensitivity fluorescence detection systems are being continuously improved.^20–22^ However, studies addressing the subcellular distribution of platinum-based anticancer drugs using X-ray fluorescence microscopy remain limited thus far to the resolution of the cytoplasmic and nuclear compartments.^23,24^ Transmission electron microscopy (TEM) with energy dispersive X-ray spectroscopy (EDS) or energy filtering (EFTEM) has been successfully used for gold subcellular imaging,^25^ and offers unparalleled lateral resolution, but limited sensitivity in case of platinum.

NanoSIMS is an advanced type of dynamic secondary ion mass spectrometer, which was designed particularly for trace element and isotope analysis with high spatial resolution (down to 50 nm probe size).^26,27^ The applicability of NanoSIMS for subcellular mapping of platinum deposited in eukaryotic cells was demonstrated by Usami *et al.*^28^ and Eybe *et al.*^29^ NanoSIMS is frequently combined with stable isotope probing (*e.g.*^13^C/^12^C and/or ^15^N/^14^N), which enables monitoring of the cellular uptake and distribution of isotopically labeled compounds.^30–35^ Some of the advantages of NanoSIMS over different visualisation techniques for metal-based complexes have already been outlined by other authors.^25,36^ In the case of platinum-based complexes, the capabilities of multi-elemental, isotope selective analysis allow information to be obtained about both the distribution of the metal and that of the isotopically labeled ligands, as recently shown for the cellular uptake of a polynuclear, ^15^N-labeled Pt compound by Wedlock *et al.*^37^ Moreover, NanoSIMS can be successfully combined with other techniques, such as electron microscopy, Raman microspectroscopy, confocal laser scanning microscopy and fluorescence *in situ* hybridization.^25,38–40^

In this paper, we report the combined application of NanoSIMS (for mapping the subcellular cisplatin distribution) and confocal laser-scanning microscopy (to identify the subcellular structures involved) in human cancer cells. The simultaneous detection of nitrogen-, phosphorus- and sulfur-containing secondary ions was utilized for correlation analysis of the distribution patterns of these biologically relevant elements with those of platinum. The excellent sensitivity of NanoSIMS for the detection of both platinum and ^15^N isotopically labeled ligands is demonstrated, and we present a data evaluation procedure that enables monitoring of the ligand to central atom stoichiometry in different subcellular compartments.

## Results and discussion

Semi-thin (300 nm) sections of resin-embedded human colon cancer cells (cell line SW480), obtained from adherent cell monolayers treated with ^15^N-labeled cisplatin for 24 h, were examined using a NanoSIMS NS50L instrument from Cameca (Paris, France). The use of this rather (but not completely) cisplatin-insensitive cell line allowed for the application of cisplatin over a wide concentration range. Cell viability was confirmed with three different methods: an MTT-based cytotoxicity assay, as well as Annexin V-FITC/PI and JC-1 assays for apoptosis induction (see ESI† for details).

One of the major challenges associated with (Nano)SIMS is sample preparation. SIMS is destructive, which means that only post-mortem analysis can be accomplished. In addition, the measurement process takes place under ultra high vacuum (UHV), which leads to the collapse of cells during sample introduction. Resin embedding is efficient for the preservation of the cellular structure; however, fixation, treatment with an ethanol series, and the infiltration of acetone–resin mixtures can lead to the diffusible fraction of the drug being washed out. Consequently, it has to be assumed that the data acquired on the resin sections mainly refer to tightly bound cisplatin.

**Fig. 1:**
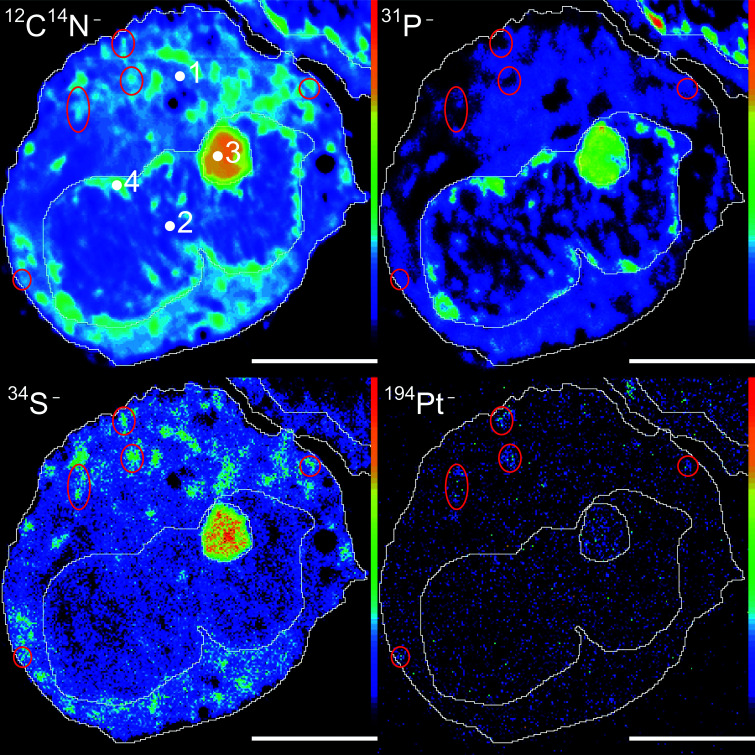
Exemplary ^12^C^14^N^−^, ^34^S^−^, ^31^P^−^ and ^194^Pt^−^ secondary ion signal intensity distribution maps acquired from semi-thin sections of SW480 cells treated with 25 μM of cisplatin. Regions of interest (ROIs) were manually defined within: (1) the cytoplasm; (2) the nucleus; (3) the nucleolus; and (4) the chromatin. According to the ^194^Pt^−^ and ^34^S^−^ signals, the drug is accumulated in small cytoplasmic, sulfur-rich aggregates (encircled in red), as well as in the nucleoli. Intensities are displayed on a rainbow false-color scale, ranging from dark blue to red for low to high intensities, respectively. The scale bars are 5 μm.

**Fig. 2:**
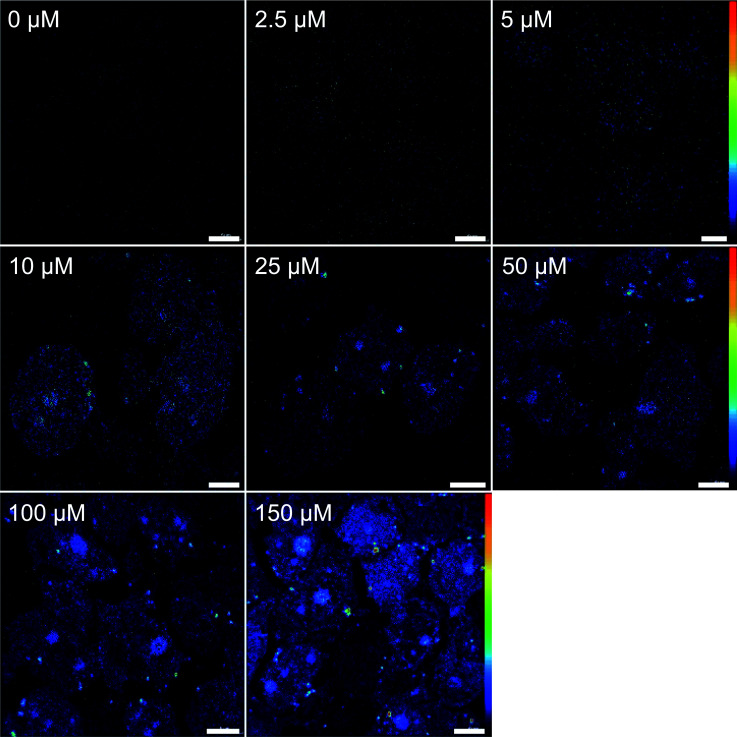
NanoSIMS ^194^Pt^−^ signal intensity distribution images obtained from semi-thin resin sections of SW480 cells treated with CDDP with various concentrations for 24 h. Signal intensities are displayed on a rainbow false-color scale, ranging from 0 counts per pixel (black) to max. 4 and 10 counts per pixel (red) for images from cells exposed to 0 to 10 μM and 25 to 150 μM CDDP, respectively. The primary ion beam was rastered over areas between 32 × 32 to 40 × 40 μm^2^ at a resolution of 512 × 512 pixels for a total dwell time of 200 to 300 ms per pixel. Scale bars = 5 μm.

Fig. 1 displays elemental distribution maps obtained from a semi-thin resin section of cells treated with 25 μM CDDP. The cellular compartments were manually defined based on characteristic features which were identifiable in the secondary ion images, as follows: (1) cytoplasm, which includes all of the cell area outside the nucleus; (2) nucleus, demarcated by the chromatin rim (likely representing heterochromatin), but excluding the nucleolus (to avoid biasing); (3) nucleolus, a round shaped C-, N-, P- and S-rich structure inside the nucleus; and (4) chromatin, indicated by dense, phosphorus-rich (but not sulfur-rich), unevenly distributed regions along the inner membrane of the nucleus (see also Fig. S1† for an untreated control, and Fig. S2† for a sample of cells treated with 150 μM cisplatin). The ^194^Pt^−^ signal intensity distribution revealed an accumulation of platinum in both the nucleic and cytoplasmic compartments. In the cytoplasm, platinum was accumulated in small aggregates, most of which were also rich in sulfur. In the nucleus, most of the platinum was associated with the nucleolus, which is rich in sulfur as well as phosphorus (Fig. 1 and S2†). The Pt distribution pattern was found to be similar for all samples exposed to ≥10 μM CDDP (for comparison, clinical peak plasma concentrations of cisplatin range between 8 and 64 μM, depending on the administration regimen);^41–43^ at lower concentrations, the signals were more diffusely distributed (Fig. 2). However, even at CDDP concentrations as low as 2.5 μM, region of interest (ROI) specific analysis of the ^12^C_2_^−^ normalized ^194^Pt^−^ signal intensities showed a significant enhancement of the average platinum signal intensity within treated cells, relative to the untreated control (Fig. S3;† Student's *t*-test, *p* < 0.002). ICPMS analyses of replicate samples revealed that the average platinum content in cells treated with 2.5 μM CDDP was <5 fg per cell (unpublished data). Taking into account that a 300 nm thick section of a cell which is 10 μm in diameter contains less than 5% of the total cell volume, the ICPMS results provide evidence of the impressive sensitivity of NanoSIMS for platinum detection in organic matrices. Among the subcellular compartments, the chromatin structures show a tendency for enhanced Pt accumulation compared to the average nucleus (without the nucleolus) and cytoplasm values, which becomes significant at exposure concentrations ≥100 μM (Fig. S3,† Student's *t*-test, *p* < 0.022). Moreover, the local accumulation of platinum within the nucleoli increased with increasing exposure concentrations, whereas the relative distribution in other compartments showed no significant changes, as evidenced by comparison of the ^12^C_2_^−^ normalized local ^194^Pt^−^ signal intensities (Fig. S4†).

DNA is supposedly the crucial target for cisplatin, and this is consistent with our results, since phosphorus-rich regions (chromatin) yielded comparable or even elevated Pt signals with respect to other cellular compartments (*e.g.* the cytoplasm and nucleus without the nucleolus) (Fig. 1 and S2†). However, basic coordination chemistry (in particular, the principle of hard and soft acids and bases) would also predict Pt binding to S-donor ligands such as thiols.^44,45^ It has been consistently postulated that intracellular sulfur-containing molecules such as glutathione, metallothioneines and thioredoxins compete with DNA for metallodrug binding.^46–48^ This is also in accordance with our findings, as high amounts of Pt were co-localised with S-rich structures (nucleoli, cytoplasmic aggregates) in the cells (Fig. 1 and S2†). The anticancer efficacy of platinum-based agents depends on the balance between the target efficiency (DNA binding) and biotransformation by sulfur nucleophiles. Furthermore, methionines have been suspected to be favorable binding sites for cisplatin on proteins,^49^ and, in particular, methionine-rich motifs (Met-motifs) of copper transporter 1 (Ctr1) were shown to effectively bind cisplatin,^50^ with subsequent ammine ligand dissociation.^51^ Metallothioneines – cytosolic thiol-rich scavenging proteins – were also shown to bind to cisplatin, hence provoking the loss of amine ligands, and playing an important role in the sequestration and detoxification of heavy metals.^46,52^ Binding to S-donors results in the trans-labilization of ligands, facilitating the release of the (otherwise inert) ammine ligands.^45,53^

**Fig. 3:**
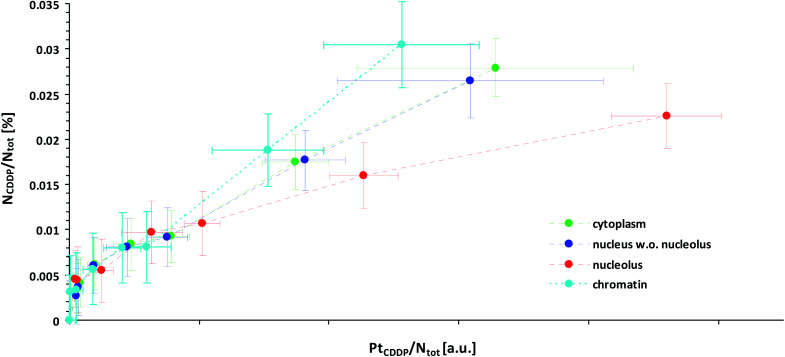
Local subcellular ligand *vs.* central atom accumulation in SW480 cells upon 24 h exposure to ^15^N-labeled cisplatin with eight different concentrations ranging from 0 μM (min. values, negative control) to 150 μM (max. values), as inferred from NanoSIMS Pt/N elemental mapping and determination of the local ^15^N/^14^N isotopic composition. Note that the slope of the curves is proportional to the N/Pt stoichiometry of the accumulated cisplatin. In particular, any flattening of the curves is indicative of cleavage and loss of ammine ligands. Symbols in green, blue, red and cyan refer to values obtained from ROIs defined within the cytoplasm, nucleus (excl. nucleolus), nucleoli and chromatin of individual cells, respectively. The error bars refer to one standard deviation calculated from the compartment specific ROI values. The mean counting error, which is an inverse measure of the analytical precision, was smaller than the dispersion of the individual ROI values (see also Fig. S7 and S8†). Further information about the calculation of the displayed quantities is provided in the text and in more detail in the ESI.†

This effect might explain our finding that Pt-enriched nucleoli deviated from the other cellular compartments in their ligand to central atom stoichiometry of the accumulated CDDP, as displayed by the different slopes in the relative Pt to N uptake curves shown in Fig. 3. It has to be emphasized that the plotting of the ^15^N isotope enrichment *versus* the platinum signal intensity, which is more intuitively appealing, fails to indicate the compound stoichiometry, since the dependency of the isotopic enrichment level on the local nitrogen content is omitted. (In such a plot, nitrogen-rich compartments exhibit lower isotopic enrichment than nitrogen-poor areas, even if equal amounts of ^15^N-labeled CDDP are accumulated, which may be misinterpreted as stoichiometric variations.) Accordingly, the secondary ion signals associated with the ligand(s) and the central atom need to be related to the total nitrogen content of the considered region. In fact, the fraction of nitrogen atoms which originate from the CDDP accumulation (N_CDDP_/N_tot_) can be obtained from the abundance of the isotopic label (^15^N/(^14^N + ^15^N)) contained in the administered compound (*a*_^15^N,CDDP_), the analysed sample (*a*_^15^N,tot_) and the untreated control (*a*_^15^N,ctr_) using an expression which reads:1
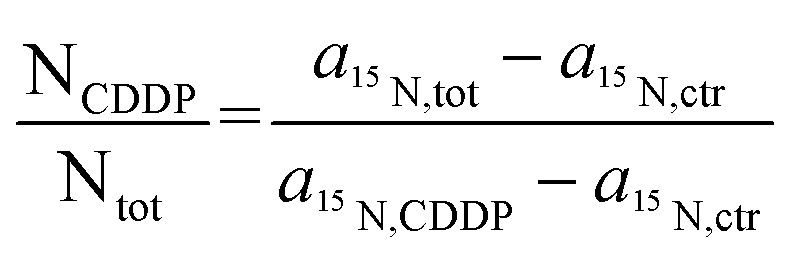


It should be noted that the relationship is quantitative and does not require any further assumptions. Fig. 3 illustrates that the maximum amount of nitrogen originating from the ammine ligands was <0.4 per mil of the total nitrogen contained in the analysed cellular compartments, which, from an analytical point of view, corroborates the high performance of NanoSIMS in spatially resolved nitrogen isotope analysis. In contrast to the isotopically labeled ligands, the relative amount of accumulated platinum cannot be directly determined from the acquired data, since SIMS is only semi-quantitative for elemental analysis. What can be defined, however, is a quantity that is proportional to the concentration ratio (Pt/N_tot_). Utilizing the ^12^C_2_^−^ secondary ion signal as a reference, this quantity can be formulated as a function of the measured ^194^Pt^−^ and ^12^CN^−^ signal intensities as follows:2
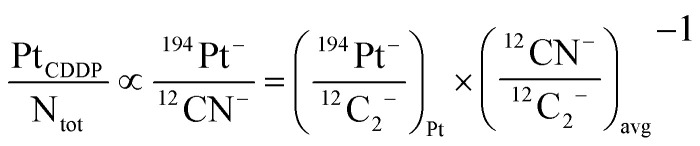
where the subscripts of the bracketed terms indicate values that were obtained from platinum measurements (‘Pt’) and averages from the platinum and ^15^N measurements (‘avg’), respectively. (Owing to instrumental constraints, the platinum distribution and nitrogen isotope composition had to be determined in sequential measurements; for details see the section ‘NanoSIMS analysis’ in the ESI.†) When considered together, the slope of the graph obtained from plotting (N_CDDP_/N_tot_) *vs.* (Pt_CDDP_/N_tot_) is proportional to the relative amounts of the accumulated ligands and central atoms. Fig. 3 shows that the nitrogen accumulation in nucleoli is disproportionally lower than that of Pt with increasing cisplatin concentrations, suggesting the cleavage of (ammine) ligands from the central atom. Moreover, the ROI analysis indicated an increase in the local sulfur content in the compartments (in particular in the nucleoli) of the cells treated with 100 μM and 150 μM CDDP (Fig. S5†), supporting the idea that N–Pt bond integrity might be affected by S-donors such as thiol-bearing molecules. In fact, we observed the colocalisation of Pt with S-rich structures; however, not all S-rich organelles are subject to cisplatin accumulation (Fig. 1 and S2†). These data suggest platinum accumulation in specific organelles, rather than being sulfur-directed or randomly distributed.

Cisplatin may enter the cytoplasm in its native state *via* active transport (including endocytosis) and/or passive diffusion. The difference in chloride concentration between the cell culture medium, which is comparable to extracellular liquids (>100 mM), and the cytosol (<20 mM) is favourable for cisplatin aquation inside the cell. In aqueous solutions at neutral pH, the chlorido ligands of cisplatin are replaced stepwise by water. The bound water molecules are very labile, so cisplatin is capable of reacting preferably with the S- and N-donor ligands in the cell. Taking into account: (a) the affinity of cisplatin for thiol-containing molecules, (b) the possible affinity for acidic pH (cisplatin accumulation in acidic organelles was previously reported^24,54^), and (c) the morphological features of the organelles (small, vesicle-like structures), we hypothesised that cisplatin may co-localise in the cytoplasm with lysosomes.

**Fig. 4:**
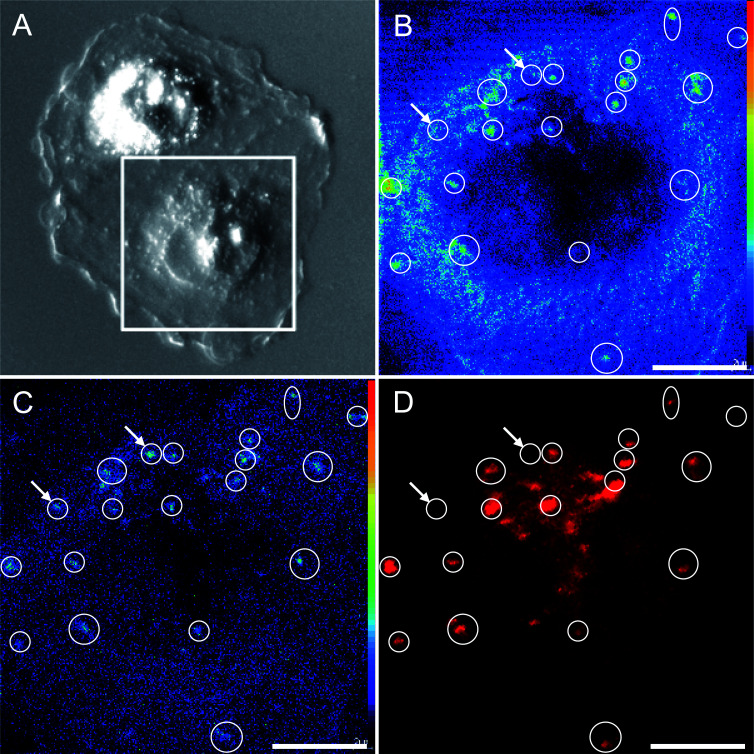
NanoSIMS secondary ion signal intensity distribution maps and confocal microscopy images of an adherent SW480 cell after 24 h exposure to 25 μM cisplatin, revealing cisplatin colocalisation with acidic organelles in the cell. The screened region is marked with a frame in the differential interference contrast image of the cell (A). The confocal microscopy image of the (fluorine-containing) LysoTracker Red (D) demonstrates a correlation with the signal intensity patterns of both ^19^F^−^ originating from LysoTracker Red (B) and ^194^Pt^−^ originating from cisplatin (C) detected by NanoSIMS in the same cell. The arrows indicate a few regions of platinum accumulation without fluorescence and ^19^F^−^ signal intensity enhancement (2 out of 20 labeled areas). Secondary ion signal intensities are displayed on a rainbow false-color scale ranging from dark blue to red for low to high intensities, respectively. The small misalignment between the fluorescence image and the secondary ion maps may originate from lens aberrations and/or cell shrinkage due to sample preparation. Scale bars = 5 μm.

The sensitivity and spatial resolution achieved with NanoSIMS multi-elemental mapping is high enough to distinguish platinum aggregation in the cytoplasm and the nucleolus without additional labeling. However, to reveal the nature of the cytoplasmic aggregates, we had to additionally apply confocal laser-scanning microscopy, which supported the co-localisation of the cytoplasmic platinum aggregates with acidic organelles (*e.g.* lysosomes) specifically marked with LysoTracker Red (Fig. 4). The hotspots in the ^194^Pt^−^ intensity distribution image refer to the loci of Pt accumulation within the cytoplasm. The ^19^F^−^ secondary ion signal intensity distribution pattern detected by NanoSIMS, together with the confocal microscopy image of the LysoTracker Red (LTR), demonstrate the correlated distribution of the fluorine-containing dye in acidic cytoplasmic organelles.

The organelles of the endocytotic and excretory pathways, *i.e.* the early and late endosomes, lysosomes and exosomes (secretory vesicles), are distributed throughout the cytoplasm; they undergo continuous maturation, transformation, fusion, and fission.^55,56^ The acidification of lysosomal and late endosomal compartments was reported to reach pH 4.5 and pH 4.8, respectively,^57^ and that of secretory vesicles reach approximately pH 5.5.^58^ Therefore, several organelles originating from a *trans*-Golgi network (TGN) are possibly involved in cisplatin accumulation. Lysosomes were shown by other researchers to have a great impact on subcellular sequestration and the detoxification of heavy metals.^59,60^ Melanosomes, with a lysosomal origin and acidic pH, were reported to trap Alexa Fluor conjugated CDDP (AF-CP) in cisplatin-insensitive MNT-1 melanotic melanoma cells, which was associated with low nuclear accumulation of the drug, whilst in the cisplatin-sensitive cell line KB-3, AF-CP was shown to accumulate in both the nucleus and cytoplasm.^24^ A different line of research showed that cisplatin resistance in ovarian carcinoma cells is linked to elevated acidity of lysosomes.^17^ A platinum-based drug bearing a fluorescent anthraquinone ligand was shown to reach the nucleus quickly (within the first 20 min to 2 h), followed by lysosomal sequestration after 24 h (whereby dissociation and different cellular trafficking of the ligands and platinum cannot be excluded). A defect in the endosomal/lysosomal acidification mechanism was shown to be a plausible contribution to cisplatin resistance in human epidermoid carcinoma cell lines.^61^ Thus, the lysosomal trafficking of cisplatin may alter the sensitivity/resistance of the cells (*e.g.*, depending on lysosomal acidification capacity).

Cytoplasmic platinum aggregation with no nuclear localisation has been observed by other authors using SIMS to study the uptake and distribution of non-cytotoxic dichloroterpyridine platinum (PtTC) in Chinese Hamster Ovary (CHO) cells after treatment with 350 μM of the compound (no ligand distribution was investigated in this study).^28^ Platinum signals correlated well with sulfur signals, which is in good agreement with our data. This supports the idea that S-rich molecules play an important role in the metabolisation of different platinum-based compounds. In contrast to our data, the study by Usami *et al.* showed for cisplatin, which was used as a control (30 μM, 5 h exposure), enhanced nuclear localisation with no pronounced cytoplasmic aggregation. Quite recently, the subcellular distribution of a ^15^N-labeled, highly-charged polynuclear platinum compound (20 μM, 1–2 h exposure) was investigated using NanoSIMS in human MCF7 breast adenocarcinoma cells.^37^ Both platinum aggregation in cytoplasmic vesicle-like structures and nucleolar enrichment were reported. However, no Pt signal was detected for cisplatin-treated samples (20 μM, 1–2 h exposure). The reason for the apparent discrepancy between these studies and our observations is most likely the different exposure time (1–2 h *vs.* 24 h exposure). Thus, the main difference between the behaviour of the polynuclear complex and that of cisplatin seems to lie in their different cellular pharmacokinetics, rather than fundamentally different subcellular distribution patterns.

## Conclusions

NanoSIMS proved to be a highly sensitive technique for subcellular imaging of Pt in cell culture samples exposed to cisplatin. Application of the drug with ^15^N isotopically labeled ammine ligands in a series of concentrations, and appropriate NanoSIMS data analysis, enabled monitoring of the N/Pt stoichiometry of the compound, which suggested partial dissociation of the Pt–N bonds, at least in nucleoli at elevated treatment concentrations. The colocalisation of platinum primarily with sulfur, but also with phosphorus-rich structures, is consistent with the known high affinity of platinum for sulfur donors, and binding to DNA as the generally accepted critical target. The indicated cleavage of ligands from platinum colocalising with S-rich nucleoli is consistent with the important role of thiol-bearing molecules (affecting Pt–N bond stability) in platinum processing. The partial dissociation of Pt–N bonds raises the question of possible implications for the mechanism of action with regard to nuclear targets. The accumulation of platinum within acidic organelles, as visualised by the combined application of NanoSIMS elemental mapping and fluorescence microscopy, might be relevant for the detoxification and/or mode of action of platinum drugs. In the case of the investigated SW480 cells, the cisplatin aggregation in S-rich acidic cytoplasmic organelles may contribute to their intrinsic resistance mechanisms. Additional experiments addressing possible parallels in Cu and Pt accumulation (due to the role of copper transporters in Pt uptake), and the use of ^36^S-labeled thiols to study their involvement in platinum processing, should be considered.

Multi-elemental imaging by means of NanoSIMS might also reveal interactions/affinities of platinum with elements other than N, P and S. The methodological approach described here should also enable the investigation of antitumour compounds based on other metals (*e.g.* Ru, Os, Ga) and/or containing different isotopic labels (*e.g.*^2^H, ^18^O, ^13^C, ^36^S), which renders NanoSIMS a powerful and widely applicable tool for subcellular drug imaging. Specifically, we have demonstrated the ability to obtain quantitative information about the distribution of the metal *vs.* ligand, shedding light on metal–ligand bond integrity. The combination of SIMS with complementary analytical and imaging techniques can aid in identifying and characterising subcellular structures highlighted in elemental distribution patterns, as exemplified in our study by applying fluorescence confocal laser scanning microscopy with intracellular markers prior to NanoSIMS measurement of the same region of interest.

## Supplementary Material

SC-005-C3SC53426J-s001
