# Supplementary material for: NanoSIMS combined with fluorescence microscopy as a tool for subcellular imaging of isotopically labeled platinum-based anticancer drugs
Source: Chem Sci. 2014 Jun 6;5(8):3135–43. doi: 10.1039/c3sc53426j (PMC9273000; doi:10.1039/c3sc53426j)
Supplement: SC-005-C3SC53426J-s001 [file SC-005-C3SC53426J-s001.pdf]

Electronic Supplementary Material (ESI) for Chemical Science.
This journal is © The Royal Society of Chemistry 2022

NanoSIMS combined with fluorescence microscopy as a tool for subcellular imaging of isotopically labeled platinum-based anticancer drugs

Anton A. Legin,[ab] Arno Schintlmeister,[c] Michael A. Jakupec,[ab] Mathea S. Galanski,[ab] Irene Lichtscheidl,[e] Michael Wagner[cd] and Bernhard K. Keppler[ab]

[a] *Institute of Inorganic Chemistry, University of Vienna, Waehringer Str. 42, A-1090, Vienna, Austria*
[b] *Research Platform "Translational Cancer Therapy Research", University of Vienna, Waehringer Str. 42, A-1090 Vienna, Austria*
[c] *Large-Instrument Facility for Advanced Isotope Research, University of Vienna, Althanstrasse 14, A-1090 Vienna, Austria*
[d] *Department of Microbiology and Ecosystem Research, Division of Microbial Ecology, University of Vienna, Althanstrasse 14, A-1090 Vienna, Austria*
[e] *Core Facility of Cell Imaging and Ultrastructure Research, University of Vienna, Althanstrasse 14, A-1090 Vienna, Austria*

Electronic Supplementary Information (ESI†)

Contents

	Experimental data	Page
Fig. S1	$^{32}S^-$, $^{194}Pt^-$, $^{12}C_2^-$ and $^{12}C^{14}N^-$ secondary ion images of a semithin section of untreated SW480 cells	3
Fig. S2	$^{12}C^{14}N^-$, $^{34}S^-$, $^{31}P^-$ and $^{194}Pt^-$ secondary ion maps of a semithin section of 150 μM CDDP treated cells	4
Fig. S3	Platinum distribution in SW480 cells as inferred from the $^{194}Pt^-$ signal intensities normalised to $^{12}C_2^-$	5
Fig. S4	Comparison of the relative platinum signal intensities ($^{194}Pt^-/^{12}C_2^-$)	6
Fig. S5	Relative subcellular sulfur distribution upon 24 h exposure to ^{15}N labeled cisplatin	7
Fig. S6	NanoSIMS mass spectra at m/z = 194 u	8
Fig. S7	Mean analytical precision (counting error) versus dispersion of ROI data ($^{194}Pt^-/^{12}C^{14}N^-$)	9
Fig. S8	Mean analytical precision (counting error) versus dispersion of ROI data ($^{12}C^{15}N^-/^{12}C^{14}N^-$)	10
Fig. S9	Effect of cisplatin on growth of SW480 cells, determined by the MTT assay (24 h exposure)	11
Fig. S10	Apoptosis/necrosis induction in SW480 cells after 24 h exposure to indicated concentrations of cisplatin	12
Fig. S11	Induction of mitochondrial membrane depolarization in SW480 cells after 24 h exposure to cisplatin	13
	**Materials and methods**	13
	Compounds	13
Scheme S1	Structures of cisplatin and platinum oxime complex **3**	14
	Cell culturing	14
	Cell viability assay	14
	Apoptosis assay	15

JC-1 assay **15**

Fluorescence microscopy **15**

Preparation of semithin sections for NanoSIMS **16**

NanoSIMS analysis **16**

Image processing, numerical data evaluation and calculations **17**

Quantities applied in data representation, stoichiometric calculations **18**

References **23**

Experimental data

NanoSIMS data

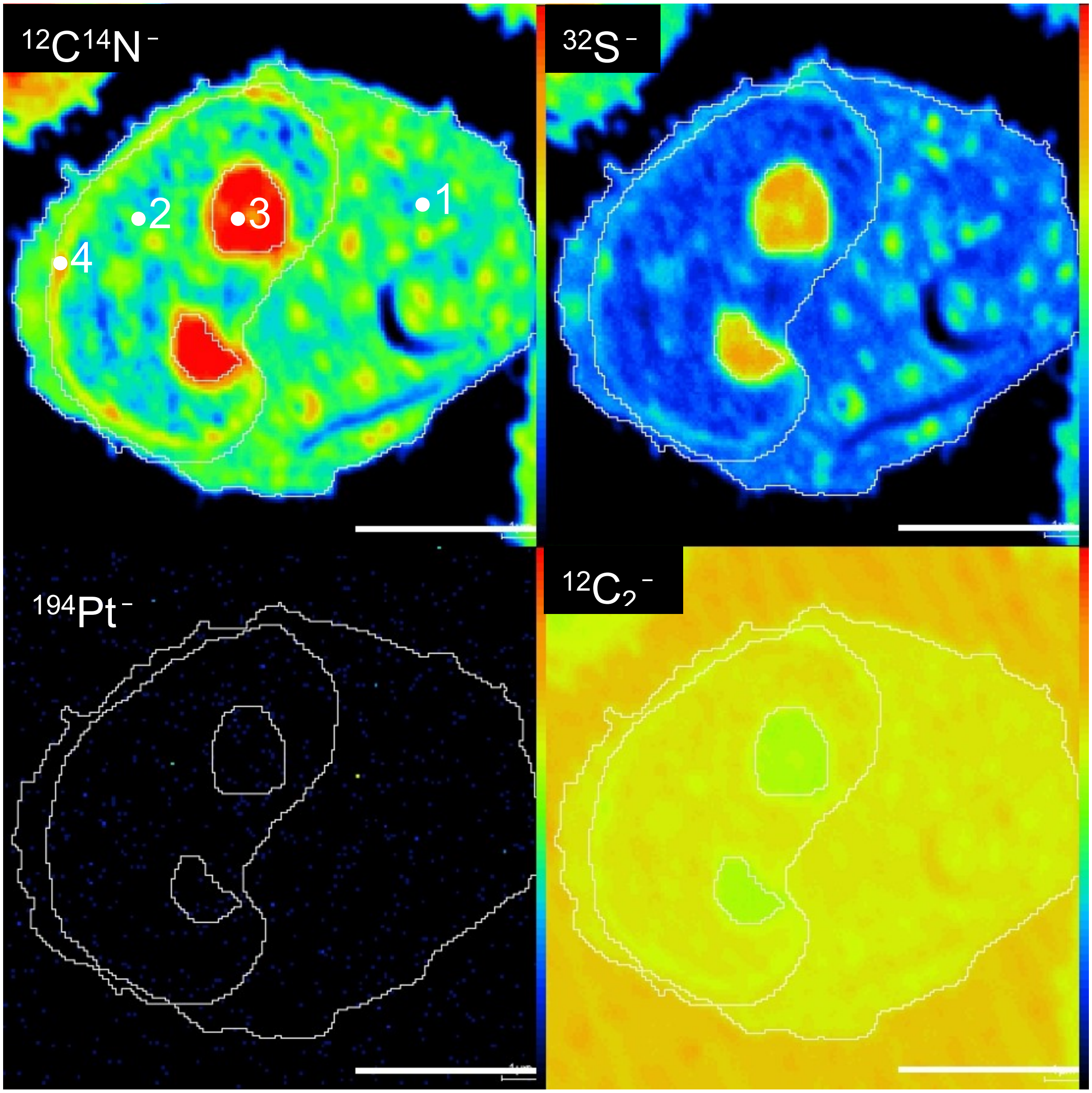

Fig. S1 NanoSIMS $^{32}S^-$, $^{194}Pt^-$, $^{12}C_2^-$ and $^{12}C^{14}N^-$ secondary ion signal intensity distribution maps of a semithin section of untreated SW480 human colon cancer cells. ROIs refer to (1) cytoplasm, (2) nucleus, (3) nucleolus, (4) chromatin. The few counts visble in the Pt channel originate from electronic noise and - to a minor degree - the detection of isobars. Intensities are displayed on a rainbow false-colour scale ranging from dark blue to red for low to high intensities, respectively. Scale bars = 5 µm.

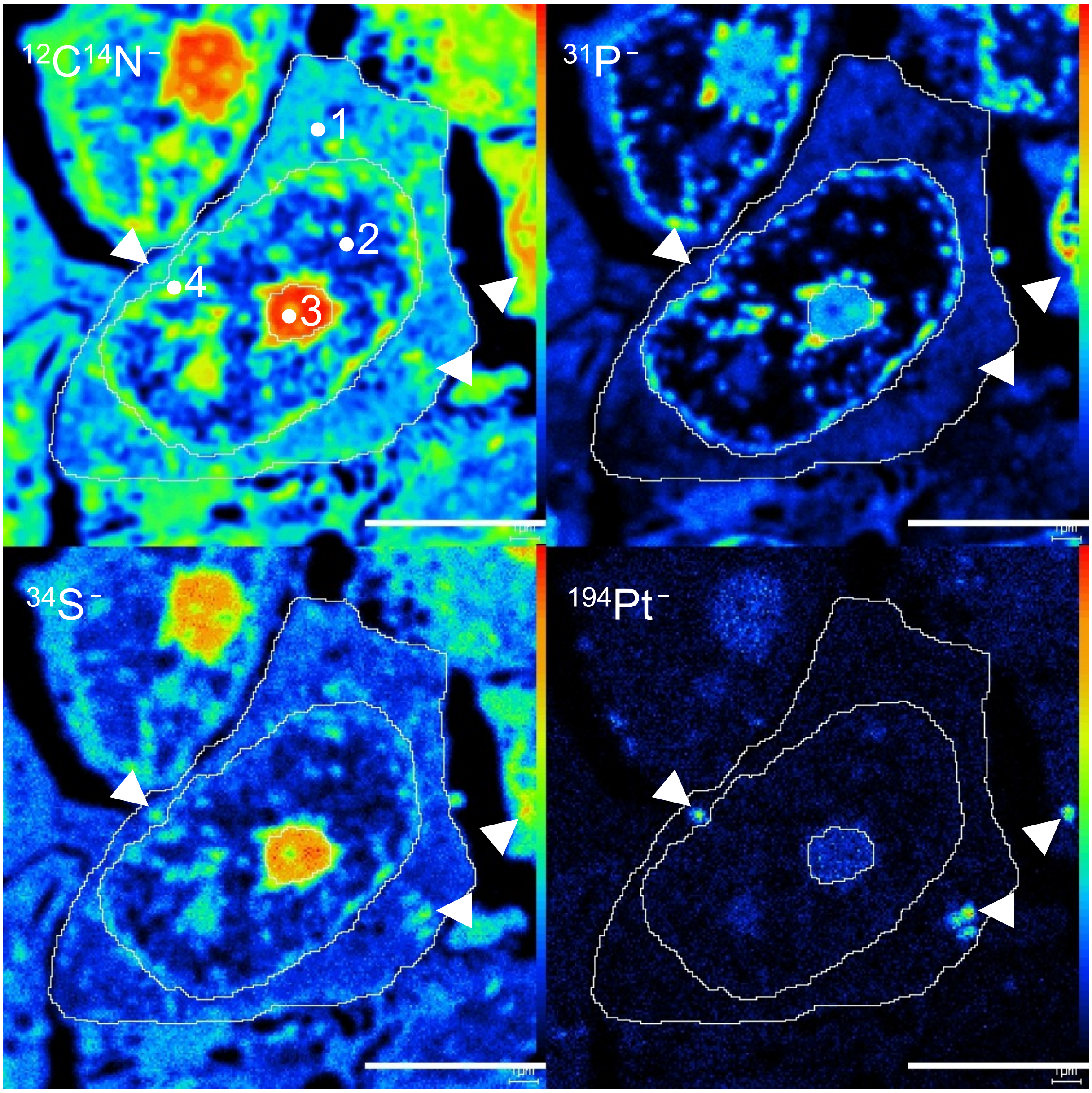

Fig. S2 NanoSIMS $^{12}C^{14}N^-$, $^{34}S^-$, $^{31}P^-$ and $^{194}Pt^-$ secondary ion signal intensity distribution maps of a semithin section of SW480 human colon cancer cells upon treatment with 150 µM CDDP for 24 h. ROIs refer to (1) cytoplasm, (2) nucleus, (3) nucleolus, (4) chromatin. Note the cytoplasmic accumulation of platinum in sulfur-rich aggregates (white arrowheads). Intensities are displayed on a rainbow false-colour scale ranging from dark blue to red for low to high intensities, respectively. Scale bars = 5 µm.

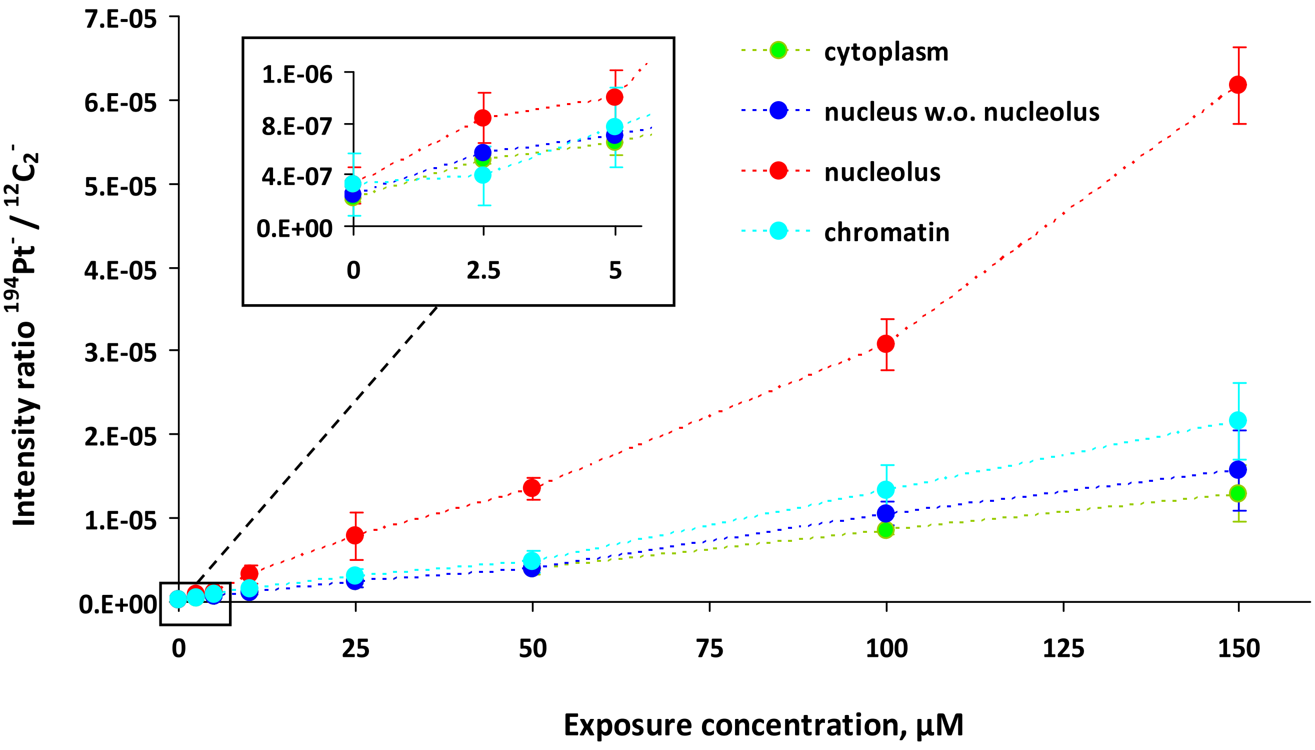

Fig. S3 Subcellular platinum distribution in SW480 cells upon 24 h CDDP exposure in the concentration range from 0 µM (min. values, control) to 150 µM (max. values), as inferred from the $^{12}C_2^-$ normalised $^{194}Pt^-$ signal intensities. The different colours of the symbols refer to values obtained from ROIs defined within the cytoplasm (green), nucleus (excl. neucleolus, dark blue), chromatin (cyan) and nucleoli (red) of individual cells. Error bars correspond to one standard deviation of the individual ROI values within each compartment. Note that the chromatin structures show a tendency of enhanced Pt accumulation in comparison to the averaged nucleus (w.o. nucleolus) and the cytoplasm, which becomes significant at exposure concentrations ≥ 100 µM (Student's t-test, $p < 0.022$).

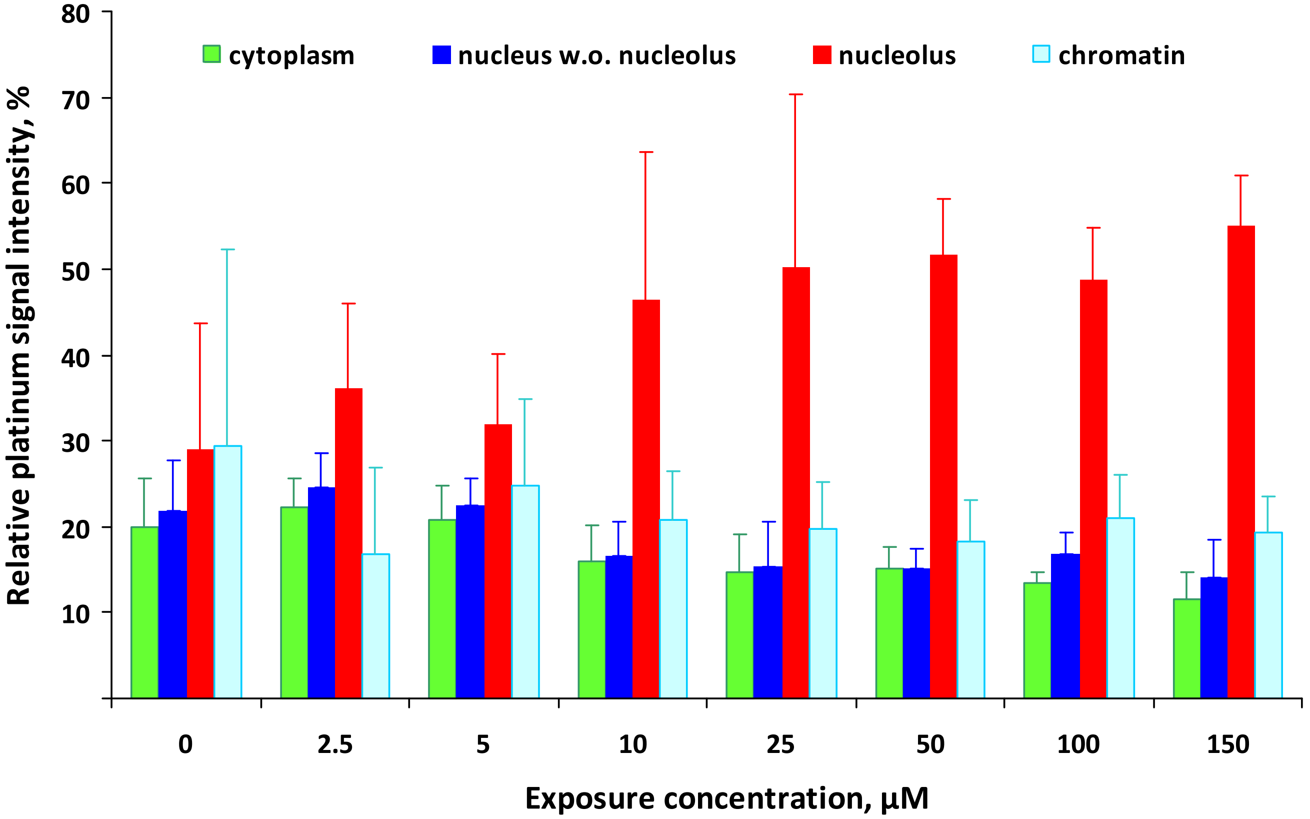

Fig. S4 Comparison of the relative platinum signal intensities ($^{194}Pt^-/^{12}C_2^-$) over the indicated compartments of SW480 cells upon 24 h CDDP exposure. 100% refers to the sum of the mean intensities for each concentration as obtained from the ROI analysis. Error bars refer to one standard deviation of the ROI values within the respective compartment.

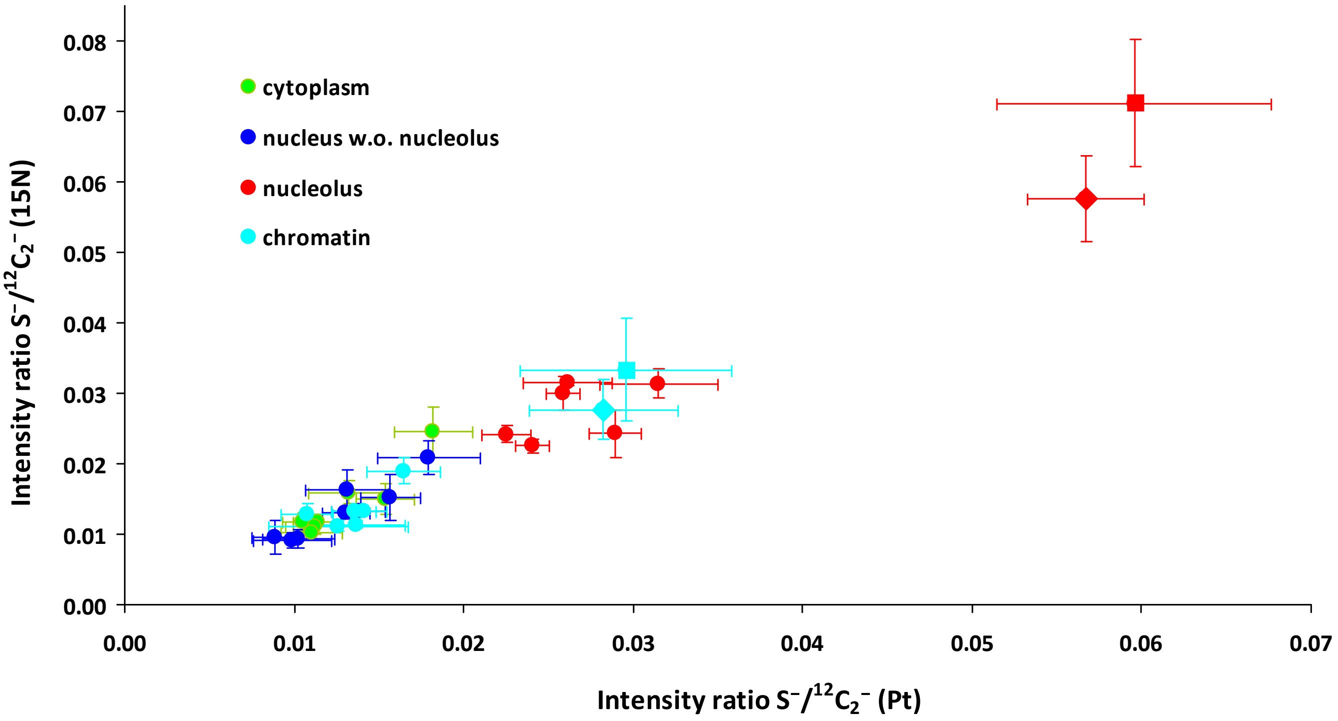

Fig. S5 Relative sulfur content of cellular compartments after 24 h exposure to eight different concentrations of cisplatin, ranging from 0 to 150 µM. as inferred from the $^{12}C_2^-$ normalised signal intensities of atomic sulfur secondary ions (S^-). Values shown on the abscissa and ordinate were acquired in the Pt and ^{15}N measurement runs, respectively. The (hypothetical) S^- signal intensities were calculated from the detected $^{32}S^-$ and $^{34}S^-$ signals by taking into account the natural abundances of the two isotopes (95.02% and 4.21%, respectively). Error bars refer to one standard deviation. Note the considerable enhancement of the sulfur content within nucleoli after treatment with 100 (squares) and 150 µM (diamonds) CDDP.

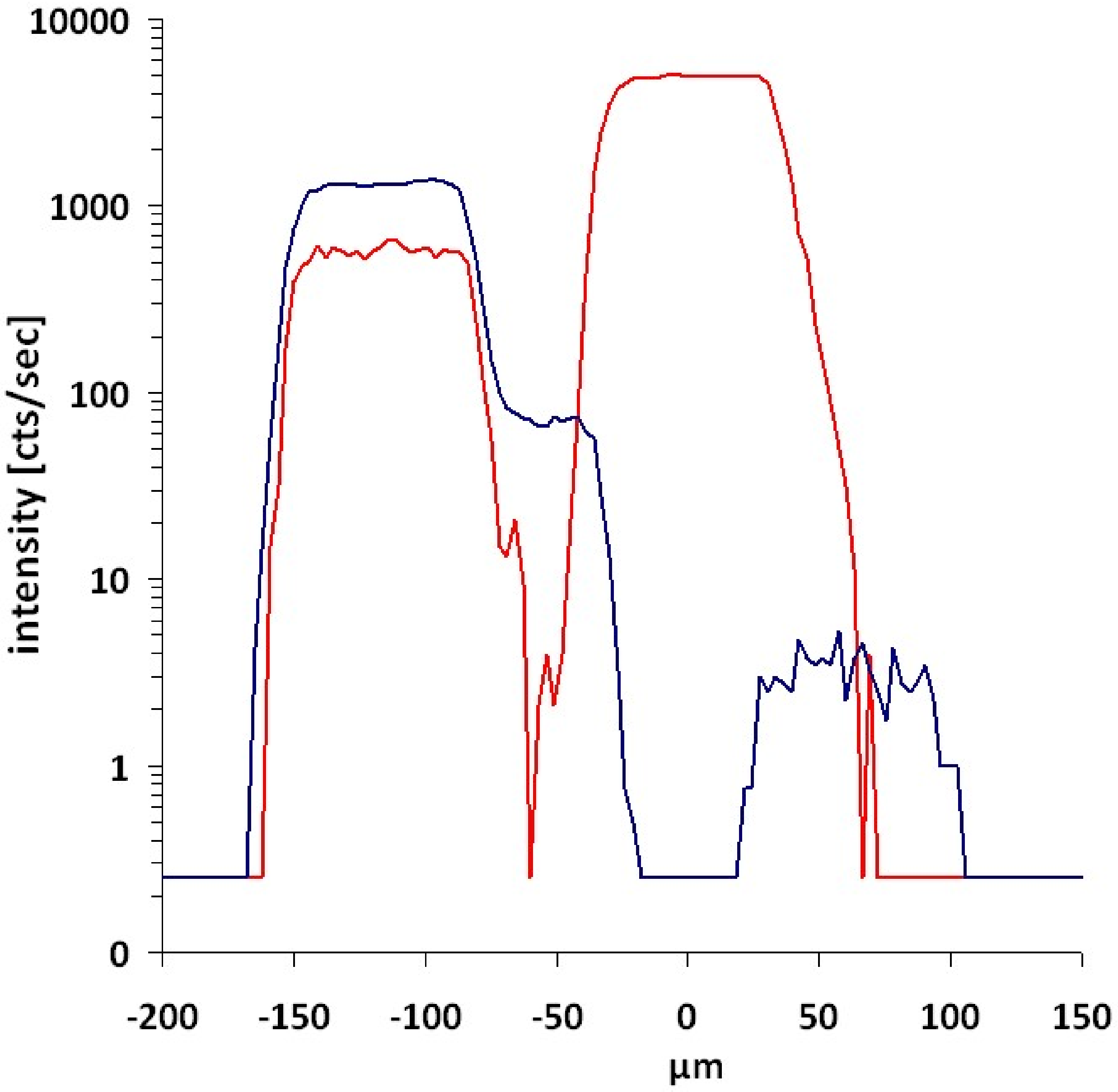

Fig. S6 NanoSIMS mass spectra at m/z = 194 u. The red curve refers to the spectrum obtained from the Pt reference sample (finely grained CDDP deposit on a semi-thin resin section of SW480 cells), the blue curve displays the spectrum of the negative control (semi-thin resin section of untreated SW480 cells).

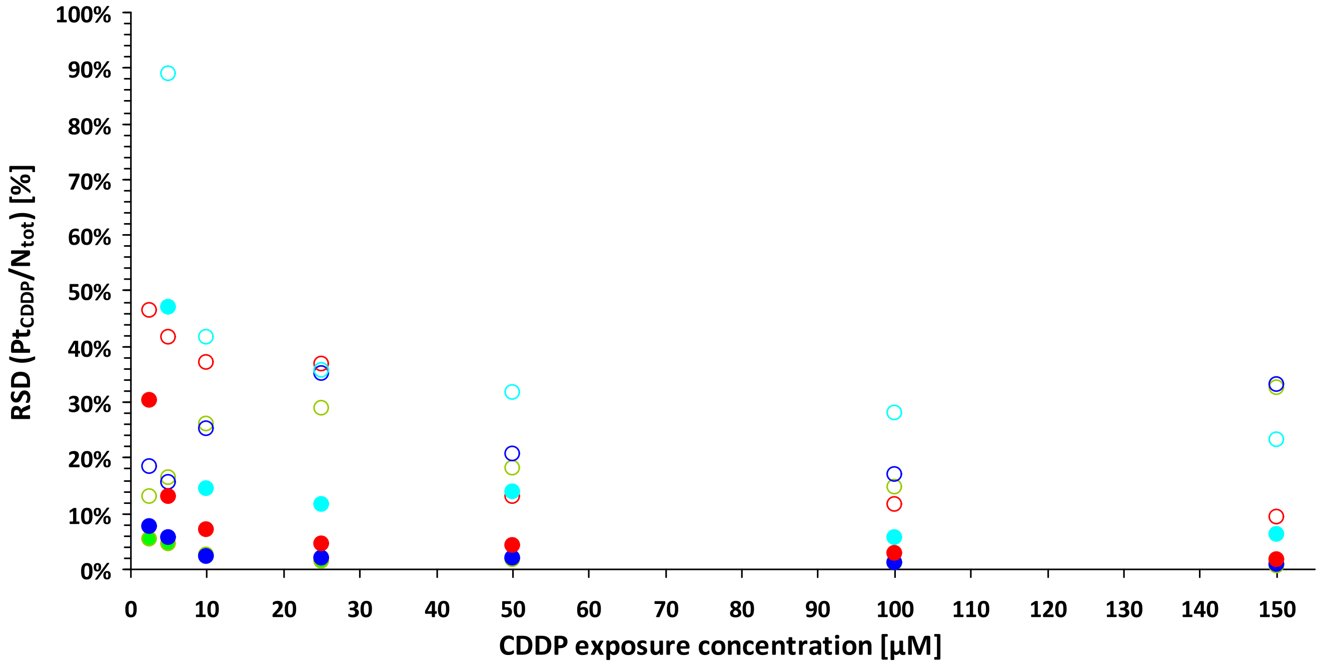

Fig. S7 Mean analytical precision (counting error, solid symbols) versus dispersion of the ROI data (open symbols) of $^{194}Pt^{-}/^{12}CN^{-}$ secondary ion signal intensities plotted on the abscissa of Fig. 3 (details are provided in the main text an in section "Stoichiometric calculations" below). Displayed values refer to one standard deviation, normalised to the mean (i.e. relative standard deviation, RSD). The different colours symbolise cytoplasmic areas (green), nuclei w.o. nucleoli (blue), nucleoli (red) and chromatin (cyan)..For further explanations see section 'Image processing, numerical data evaluation and calculations' in ESI).

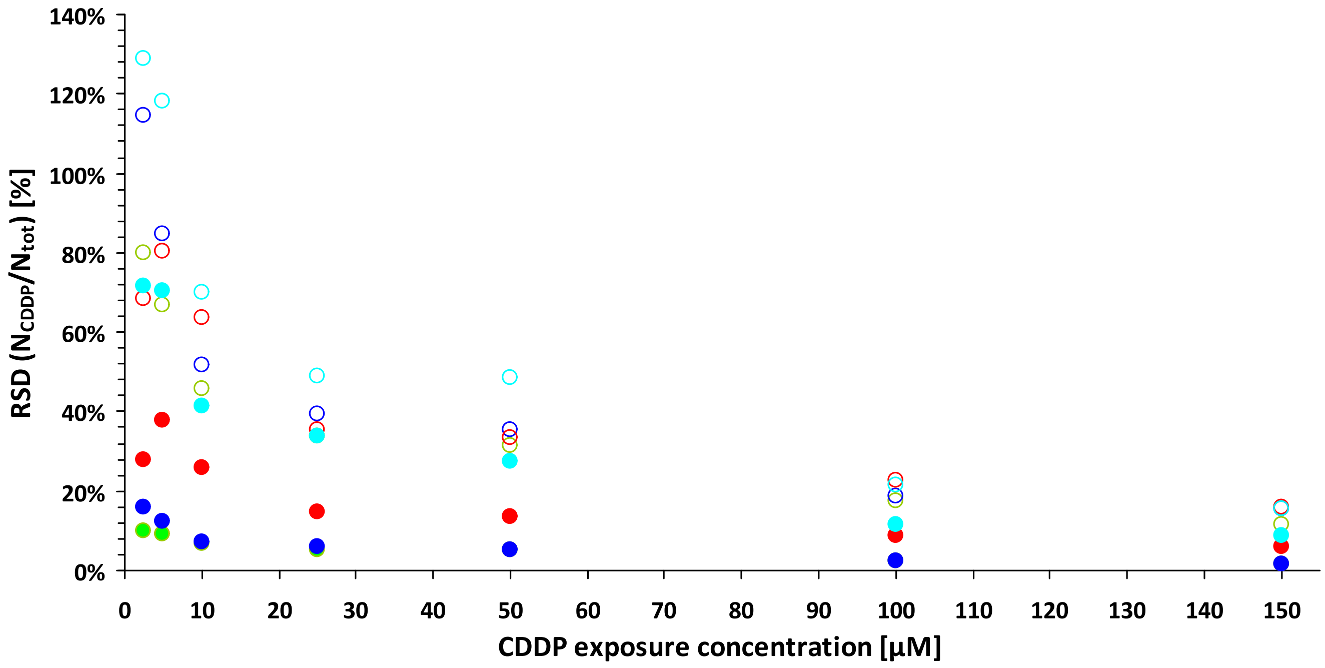

Fig. S8 Mean analytical precision (counting error, solid symbols) versus dispersion of the ROI data (open symbols) for the fraction of nitrogen atoms originating from the ammine ligands (N_{CDDP}/N_{tot}), plotted on the ordinate of Fig. 3) as inferred from the ^{15}N isotope abundance in the sample, the applied CDDP and an untreated control (see Eq. 1 in the main text, details are provided in section "Stoichiometric calculations" below). Displayed values refer to one standard deviation, normalised to the mean (i.e. relative standard deviation). The different colours symbolise cytoplasmic areas (green), nuclei w.o. nucleoli (blue), nucleoli (red) and chromatin (cyan).

Effect of cisplatin on cell viability

In order to verify that the applied CDDP concentrations are not causing cell death to a high extent within the time frame of the experiment, viability of the cells after 24 h exposure was tested with three different assays. Information about cell viability is crucial because the likelihood of cisplatin distribution artifacts will increase in dead cells due to membrane damage and breakdown of transport mechanisms.

In the MTT assay, the growth of SW480 colon carcinoma cells treated with cisplatin is not completely inhibited at concentrations lower than 100 µM (Fig. S9). The concentration of 100 µM causes total growth inhibition (complete cytostasis). The cell number is only diminished in comparison to the cell number at the beginning of exposure upon treatment with concentrations of 150 µM and higher. These observations confirm that SW480 cells are rather insensitive to cisplatin during 24 h exposure period.

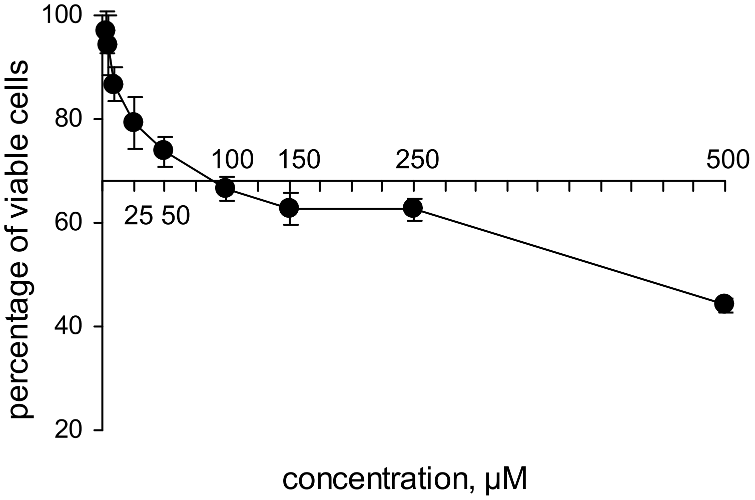

Fig. S9 Concentration-dependent effect of cisplatin on growth of SW480 cells, determined by the MTT assay (24 h exposure). The intersection of the axes corresponds to the percentage of cells already present in the beginning of drug exposure. Concentrations below 100 µM do not completely inhibit cell growth; concentrations higher than 100 µM induce a gradual cell loss.

In order to verify the low percentage of dead cells upon drug exposure in our experiments with a second method, growing SW480 cultures were treated with the CDDP in various concentrations (10–500 µM) for 24 h, then double-stained with annexin V-FITC/propidium iodide and analyzed by fluorescence-activated cell sorting (FACS). This method allows to discriminate (a) necrotic cells stained with propidium iodide (cell membrane damage is a precondition for PI penetration and intercalation into DNA, yielding red fluorescence); (b) early apoptotic cells stained with annexin V-FITC (annexin V binds to phosphatidylserine, which is a marker for early apoptosis, FITC yields green fluorescence); and (c) late apoptotic cells stained with both dies from (d) viable unstained cells. Cells treated with compound **3** (Scheme S1), which was previously shown to induce apoptosis in SW480 cells within 24 h to a higher extent, were used as a positive control. Results show that concentrations of up to 150 µM of cisplatin do not induce cell death in SW480 cells within the chosen exposure time (Fig. S10).

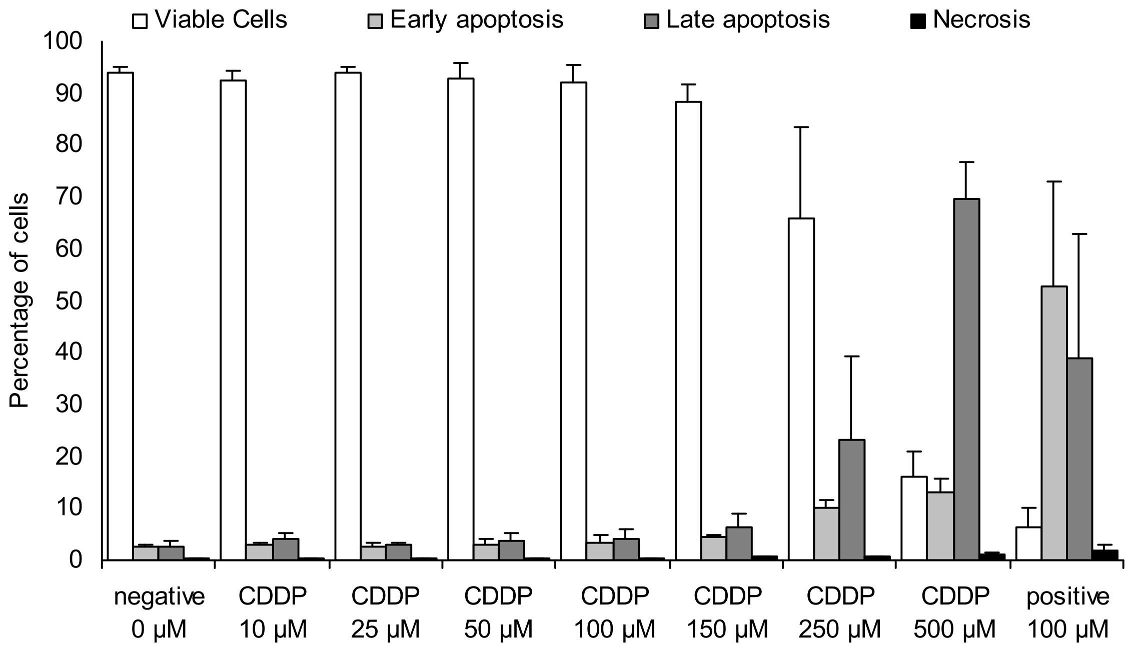

Fig. S10 Apoptosis/necrosis induction in SW480 cells after 24 h exposure to the indicated concentrations of cisplatin (CDDP), measured by FACS using annexin V-FITC/propidium iodide double-staining. Negative – untreated control, positive – cells treated with compound **3**.

In addition, mitochondrial membrane potential (MMP) was assessed by the lipophilic cationic probe JC-1, a compound selectively concentrated in intact mitochondria to form J-aggregates emitting green fluorescence, whereas depolarization of mitochondrial membranes leads to orange fluorescence of the dye, which can be measured by FACS. MMP loss is an early event in apoptotic cell death. Results confirm that cisplatin induces a marked loss of the mitochondrial transmembrane potential only in concentrations higher than 150 µM (Fig. S11).

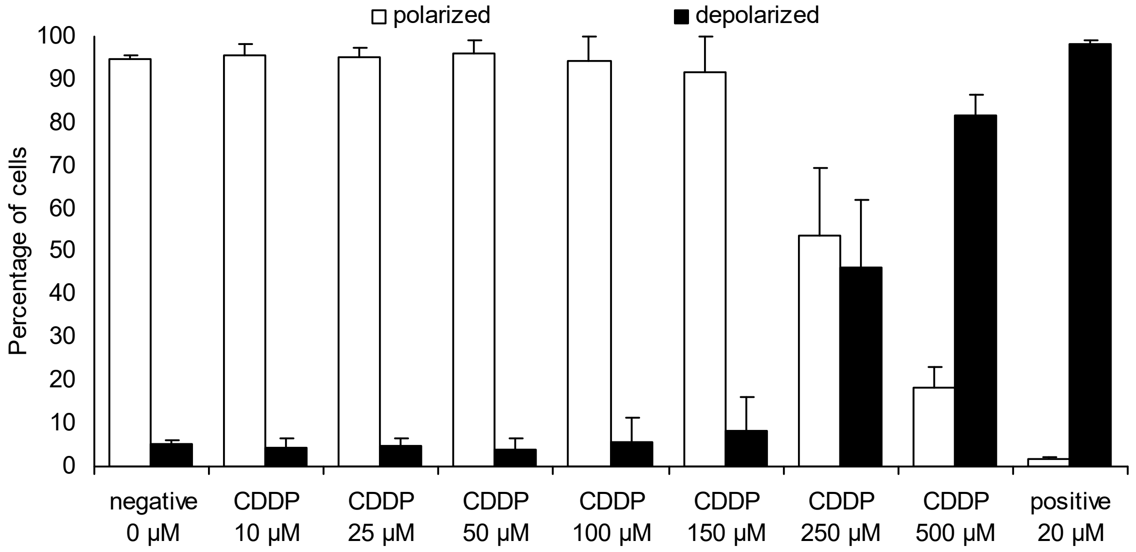

Fig. S11 Induction of mitochondrial membrane depolarization in SW480 cells after 24 h exposure to the indicated concentrations of cisplatin, measured by FACS using JC-1 staining. Negative – untreated control, positive – cells treated with CCCP (carbonyl cyanide 3-chlorophenylhydrazone).

Taken together, all three assays support the presumption that the cisplatin-treated cells that were subject to NanoSIMS measurements were neither apoptotic nor necrotic before fixation.

Materials and methods

Compounds. ^{15}N-labeled cisplatin (Scheme S1) was synthesised according to a literature procedure by using ^{15}N-labeled ammonium chloride (>98% ^{15}N enrichment level, CORTEC, USA).[1] (SP-4-1)-Dichloridobis((1*Z*)-*N*-hydroxypropane-1-imine-κ*N*)platinum(II) (complex **3**, Scheme S1) used as a positive control in the apoptosis assay was prepared according to a previously published procedure[2].

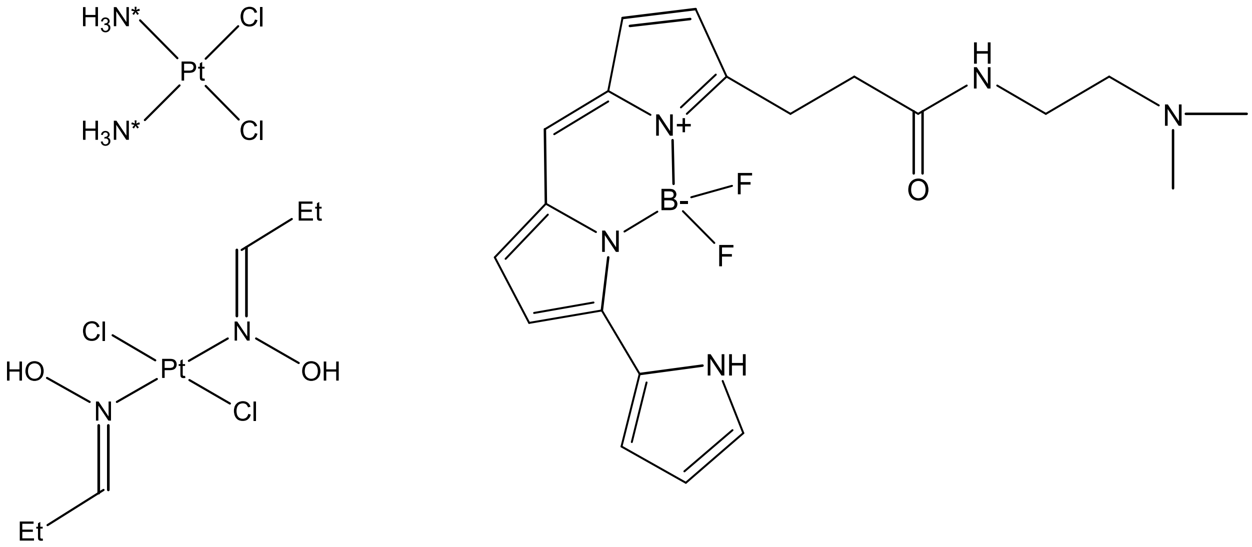

Scheme S1. Structures of cisplatin (upper left), platinum oxime complex **3** (lower left) and LysoTracker® Red DND-99 (right). ^{15}N-labeled ammine ligands are marked with asterisks.

Cell culturing. The human cancer cell line SW480 (colon carcinoma) was grown in 75 cm^2 culture flasks (Iwaki/Asahi Technoglass, Gyouda, Japan) as adherent monolayer cultures in complete medium [i.e., Minimal Essential Medium (MEM) supplemented with 10% heat-inactivated fetal bovine serum, 1 mM sodium pyruvate, 4 mM L-glutamine, and 1% nonessential amino acids solution from 100× ready-to-use stock (all purchased from Sigma-Aldrich Austria)]. Cell cultures were incubated at 37 °C in a moist atmosphere containing 5% CO_2.

Cell viability assay. The cytotoxic activity of cisplatin was determined by using the colourimetric MTT (3-(4,5-dimethyl-2-thiazolyl)-2,5-diphenyl-2*H*-tetrazolium bromide) assay to test the viability of the cells after 24 h exposure to different concentrations of the drug. SW480 cells were collected from culture flasks by trypsinization, and 1.5×10^4 cells/well were seeded in 100 µl aliquots in complete medium into 96-well microculture plates (Iwaki/Asahi Technoglass, Gyouda, Japan). The cells were allowed to settle overnight. Cisplatin was dissolved in complete medium, serially diluted, and 100 µl of the dilutions were added per well. After 24 h exposure, drug solutions were replaced with 100 µl of RPMI1640 culture medium (supplemented with 10% heat-inactivated fetal bovine serum and 2 mM L-glutamine) mixed with 20 µl MTT solution in phosphate-buffered saline (5 mg/ml). After incubation for 4 h, the RPMI/MTT mixtures were removed, and the formazan crystals formed in viable cells were dissolved in 150 µl of DMSO per well. Optical densities were measured at 550 nm with a microplate reader (ELx808 Absorbance Microplate Reader, Bio-Tek, USA). Unspecific absorption was corrected by using a reference wavelength

of 690 nm. Evaluation of growth inhibition is based on means from at least three independent experiments, each comprising six replicates per concentration level.

Apoptosis assay. In order to rule out apoptotic and necrotic cell death induction by cisplatin, cells were analyzed by fluorescence-activated cell sorting (FACS) using fluorescein isothiocyanate (FITC)-conjugated annexin V (BioVision, USA) and propidium iodide (PI; Fluka) staining. SW480 cells were seeded into 12-well plates (CytoOne, Starlab, UK) in densities of 2×10^5 cells per well in complete medium and allowed to settle for 24 h. The cells were exposed to different concentrations of cisplatin for 24 h at 37 °C. After incubation, the cells were gently trypsinised, collected in 1.5 ml tubes, washed with PBS and resuspended with FITC-conjugated annexin V (0.25 µg/ml) in binding buffer (10 mM HEPES/NaOH pH 7.4, 140 mM NaCl, 2.5 mM CaCl2) at room temperature for 15 min. PI (1 µg/ml) was added shortly before the measurement. Stained cells were analyzed with a Guava 8HT EasyCyte flow cytometer (Merck Millipore, Guava, USA) using InCyte software. Three independent experiments were conducted, and 5000 cells were analyzed in each run.

JC-1 assay. Monolayers of human SW480 colon cancer cells were seeded into 12-well plates in numbers of 1×10^5 cells/well in complete MEM. After 24 h preincubation, cells were treated with cisplatin solutions for 24 h. After exposure, the cells were trypsinised and collected in 1.5 ml tubes. The cells were washed with warm PBS before incubation with complete MEM containing 2 µM JC-1 (5,5′,6,6′-tetrachloro-1,1′,3,3′-tetraethylbenzimidazolcarbocyanine iodide, BioVision, USA) for 15 min at 37 °C. The positive control sample was incubated simultaneously with 2 µM JC-1 and 1 mM of the ionophore carbonyl cyanide 3-chlorophenylhydrazone (CCCP) for 15 min at 37 °C. Following JC-1 incubation, the cell pellets were washed with warm PBS, centrifuged and suspended in 0.5 ml PBS prior to FACS measurement (as described above).

Fluorescence Microscopy. For colocalization experiments, SW480 cells were seeded on indium tin oxide (ITO) coated glass slides (Praezisions Glas & Optik GmbH, Iserlohn, Germany) in 6-well plates (2×10^4 cells per well in 2 ml of MEM) and allowed to attach overnight. The cells were treated with 25 µM of ^{15}N-labeled cisplatin (24 h) and stained with LysoTracker Red (5 min, 500 nM). The cells were analyzed immediately after staining under fixing solution (2.5% glutaraldehyde in 0.1 M sodium cacodylate buffer). LysoTracker Red (Life Technologies, Paisley, UK) distribution was visualised with a confocal laser scanning microscope Leica SP2 (ICS Leica Microsystems, Wetzlar, Germany) equipped with 20× and 40× dry plan apo objectives. The excitation time was kept as short as possible to minimise the bleaching effect,

which gets stronger during the fixation procedure. Fluorescence was observed under blue light irradiation (488 nm) with an Ar laser. Stacks of 10 consecutive images (1 µm steps) were taken and processed with Leica Microsystems software to generate a maximum projection image. To keep the samples suitable for NanoSIMS application after microscopy, samples were washed in 0.1 M sodium cacodylate buffer to remove the fixation solution and dried on air. The dried samples were then subjected to NanoSIMS measurements. The confocal microscopy and NanoSIMS images were correlated by using GIMP software.

Preparation of semi-thin sections. In addition to dried whole cells (see above), semi-thin sections of resin embedded cells were prepared. For this purpose, SW480 cells were grown in 90 mm cell culture dishes to about 70% confluence. Cells treated with cisplatin (2.5 to 150 µM, 24 h) and untreated control cells were washed with PBS, harvested by trypsinization and pelleted in 1.5 ml tubes. Subsequently, the pellets were carefully handled through the steps of fixation with 2.5% glutaraldehyde solution for 2 h at room temperature, washing with 0.1 M sodium cacodylate buffer and dehydration in an ascending ethanol series (30%, 50%, 70%, 90% and 100% ethanol, 10 min each). 100% ethanol was then replaced with pure acetone, which was substituted gradually with low viscosity resin (100 Low Viscosity Resin, Agar Scientific, UK). Pellets in liquid resin were placed in a mold and hardened by oven heating for 18 h at 65 °C. The resin blocks were cut using a Leica microtome and freshly prepared glass knifes to obtain semi-thin sections with 300 nm thickness. For each treatment concentration, 5 to 10 sections were placed on a drop of bidistilled water on boron-doped silicon wafers (7 x 7 x 0.75 mm^3, Active Business Company GmbH, Brunnthal, Germany) and dried on a warm plate. The adherent sections were subsequently analyzed with NanoSIMS.

NanoSIMS analysis. NanoSIMS measurements were carried out on a NS 50L instrument from Cameca (Paris, France). Due to the limited length of the mass spectrometer (viz. the magnet of the double focusing electrostatic/magnetic sector field mass analyzer) and the physical width of the secondary ion detectors, the platinum distribution and the nitrogen isotope composition had to be determined in sequential analysis runs.

In the platinum measurements, the detectors of the multicollection assembly were positioned to enable parallel detection of $^{12}C_2^-$, $^{12}C^{14}N^-$, $^{31}P^-$, $^{34}S^-$ and $^{194}Pt^-$ secondary ions. With respect to sulfur it should be noted that the $^{34}S^-$ ion signal had to be chosen instead of the more intense $^{32}S^-$ signal (the natural isotopic abundance of ^{32}S and ^{34}S is 95.02% and 4.21%, respectively) since the flight path radius of $^{32}S^-$ ions was too close to the radius of $^{31}P^-$ ions for simultaneous sulfur and phosphor detection. The inner width of the selected spectrometer entrance slit was 20 µm ("ES#3") and a 200 µm aperture slit

("AS#3") was inserted in the secondary ion beam path to reduce beam divergence. The electrostatic lenses and deflectors inside the spectrometer were adjusted to achieve a mass resolving power (MRP) of 10.700 (according to Cameca`s definition) at the position of the $^{194}Pt^{-}$ detector. Spectrometer tuning, mass calibration and detector (electron multiplier) calibration were carried out on a semi-thin section of resin-embedded SW480 cells after precipitation of a finely grained CDDP deposit obtained from spotting and evaporation of an aqueous, 1 mM cisplatin solution on the sample surface. A 40 µm spectrometer exit slit ("ExS#2") was selected at the $^{194}Pt^{-}$ detector to facilitate efficient blanking of isobars, which appear both at the low and the high mass end of the peak (Fig. S6, ESI).

In the nitrogen isotope measurements, the detectors were positioned for simultaneous registration of $^{12}C_2^{-}$, $^{12}C^{14}N^{-}$, $^{12}C^{15}N^{-}$, $^{31}P^{-}$ and $^{32}S^{-}$ secondary ions. The spectrometer settings were analogous to the platinum measurements; however, the voltage at the quadrupole lens ("Q") that links the electrostatic with the magnetic sector of the spectrometer was slightly varied to achieve a MRP of 10.500 for detection of CN^{-} ions.

All data were acquired as multilayer image stacks obtained by sequential scanning of a finely focused Cs^{+} primary ion beam over areas between 30 × 30 and 40 × 40 µm^2 with 512 × 512 pixel image resolution. The physical resolution (probe size) was approx. 80 nm, accomplished through beam divergence reduction by insertion of a diaphragm with 100 µm inner diameter ("D1#3") in the primary ion beam path. The per pixel dwell time of the primary ion beam was in the range from 7.5 to 10 ms and the number of image (scanning) cycles was chosen to achieve a total dwell time of 200 to 300 ms per pixel. Between every image cycle, secondary ion beam drift was corrected by automatic beam centering and coaxial lens ("EOS") voltage optimization - utilizing the $^{12}C2^{-}$ signal as reference - as well was automatic peak centering for each of the recorded secondary ion species. The total acquisition time was in the range from 15 to 20 h per measurement. Prior to data acquisition, analysis areas were pre-sputtered by scanning of an unfocused, high intensity (400 pA) Cs^{+} primary ion beam over an area of 60 × 60 µm to remove surface adsorbates and to benefit from chemical secondary ion yield enhancement through reactive primary ion implantation. On the resin sections pre-sputtering was finished after sample irradiation with 8.3E16 ions/cm^2, which was high enough for reproducibly reaching the steady state signal intensity regime.

Image processing, numerical data evaluation. NanoSIMS image data were evaluated using the WinImage software package provided by Cameca. Prior to stack accumulation, the individual images were aligned to compensate for positional variations arising from primary ion beam and/or sample stage drift. Secondary ion signal intensities were dead time corrected on a per-pixel basis. Quasi simultaneous

arrival (QSA) effects were taken into account for C_2^- and CN^- secondary ions. In contrast to dead time correction, QSA correction had to be performed manually on a per ROI basis.

Individual regions of interest (ROIs) representative for distinct cellular compartments were defined manually, based on the morphological features identifiable in the relative N, P and S elemental distribution, as inferred from the $^{12}C^{14}N^-$, $^{31}P^-$ and $^{32}S^-$ or $^{34}S^-$ signal intensity distribution maps. In the measurements on the resin sections, the raster size, pixel number and probe size were chosen to enable simultaneous analysis of several individual cells in one measurement (see e.g. Fig. 2). Nevertheless, data were only evaluated from cells containing a complete set of the considered subcellular compartments, in particular nucleoli. Results are presented as arithmetic means and standard deviations calculated from the individual ROI data for each of the defined cellular compartments. The analytical precision was estimated from the statistical error (counting error) of the secondary ion signal intensities. Based on Poisson statistics, the standard deviation of a secondary ion signal is equal to the square root of the number of detection events (counts) recorded by the detection system, which may be written as

$$\sigma_{A^{+/-}} = \sqrt{N_{A^{+/-}}} \ , \qquad \text{* MERGEFORMAT (1.1)}$$

where $A^{+/-}$ designates the detected secondary ion species. Accordingly, the relative standard deviation (coefficient of variance) is given by $\frac{1}{\sqrt{N_{A^{+/-}}}}$. Counting errors were calculated for all secondary ion signals (per ROI) and propagated to the quantities applied in data representation (see also section ”Stoichiometric calculations” below).

As already stated above, secondary ion signal intensities were also corrected for quasi-simultaneous arrival (QSA) of secondary ions at the detector. QSA arises from the quasi-simultaneous emission of more than one secondary ion per primary ion impact and leads to counting losses in single ion counting by electron multipliers[3, 4]. It is worthy to note that QSA events are not considered in the conventionally applied dead time correction, which takes into account counting losses due to multiple secondary ion arrivals within the detector dead time (which is electronically fixed at 44 ns for the electron multiplies of our NanoSIMS NS50L). Assuming Poisson statistics, Slodzian et al. suggested a formula for QSA correction in a first order approximation [3, 4], which reads as

$$N_{cor} = N_{exp}(1 + \frac{1}{2}K) \qquad \text{* MERGEFORMAT (1.2)}$$

where $N_{exp.}$ and $N_{cor.}$ refer to the experimentally observed number of counts and the actual number of secondary ions arriving at the detector, respectively. K designates the number of ions arriving at the detector per impacting primary ion, which can be evaluated from the ratio of the secondary ion signal

intensity over the primary ion beam intensity. Since the actual number of secondary ions that reach the multiplier is unknown, K needs to be corrected via[5]

$$K_{cor} = \frac{K_{exp}}{1 - \frac{K_{exp}}{2}}$$ * MERGEFORMAT (1.3)

It needs to be emphasised that Slodzian et al. demonstrated that the multiplication factor appearing at K in Eq.* MERGEFORMAT (1.2) may deviate from the theoretical value of ½ [5]. Consequently, we conducted measurements on biomass (dried yeast cells, data not shown) which yielded multiplication factors of 1,06+/-0,04 and 1,05+/-0,02 (1σ) for C_2^- and CN^- secondary ions, respectively. Using these factors, the C_2^- and CN^- secondary ion signal intensities were QSA corrected by application of Eqs. * MERGEFORMAT (1.2) and * MERGEFORMAT (1.3). The primary ion beam intensities during the measurements were estimated from the averaged primary ion beam current measured automatically at the faraday cup in the upper part of the primary ion column ('FCp') before and after every measurement and periodical calibration of the FCp values versus the beam current measured at the faraday cup positioned behind the sample stage ('FCo').

Quantities applied in data representation, stoichiometric calculations.

$\mathbf{a_{15N}}$ refers to the abundance of the ^{15}N isotope in a given sample. Since nitrogen comprises only two stable isotopes (i.e. ^{14}N and ^{15}N), the ^{15}N abundance corresponds to the isotope fraction $^{15}N/(^{14}N+^{15}N)$, given in at%. In SIMS, the nitrogen isotope composition is inferred from the signal intensities of CN^- ions due to the high ion yield of cyanide. In this work, the ^{15}N abundance was calculated from the (QSA corrected) $^{12}C^{14}N^-$ and $^{12}C^{15}N^-$ signal intensities by

$$a_{15N} = \frac{12C15N^-}{12C14N^- + 12C15N^-}.$$ * MERGEFORMAT (1.4)

Via error propagation of the counting error, the analytical precision (1σ) achieved in an abundance measurement can be estimated from the recorded signal intensities by

$$\sigma_{a_{15N}} = \frac{1}{\left(12C14N^- + 12C15N^-\right)^2}\sqrt{\left(12C14N^-\right)^2 12C15N^- + 12C14N^-\left(12C15N^-\right)^2}.$$ * MERGEFC

Note that the signal intensities have to be applied as the (absolute) number of counts registered within the considered analysis region.

$\mathbf{R_{15N/14N}}$ refers to the ratio of $^{15}N/^{14}N$ atoms, inferred from the intensity ratio of the $^{12}C^{15}N^-$ and $^{12}C^{14}N^-$ signals. $R_{15N/14N}$ is related to the ^{15}N isotope abundance (a_{15N}) via

$$a_{15N} = \frac{R_{15N/14N}}{R_{15N/14N}+1} \text{ and } R_{15N/14N} = \frac{a_{15N}}{1-a_{15N}}. \quad \text{* MERGEFORMAT (1.6)}$$

By error propagation of the counting errors, the analytical precision (1σ) can be estimated from the recorded signal intensities (given in counts) as

$$\sigma_{R_{15N/14N,\,POISSON}} = \frac{1}{12C14N^-}\sqrt{12C15N^- + \frac{\left(12C15N^-\right)^2}{12C14N^-}}, \text{* MERGEFORMAT (1.7)}$$

N_{CDDP} / N_{tot} designates the quantity that expresses the fraction of elemental nitrogen atoms (i.e. sum over all isotopes) originating from the ligands *(N_{CDDP})* within the total number of nitrogen atoms *(N_{tot})* contained in the considered analysis region and can be calculated from the ^{15}N isotopic abundances *(a_{15N}) as*

$$\frac{N_{CDDP}}{N_{tot}} = \frac{a_{15N,tot} - a_{15N,ctr}}{a_{15N,CDDP} - a_{15N,ctr}}, \qquad \text{* MERGEFORMAT (1.8)}$$

where $a_{tot.}$ and a_{ctr} refer to the ^{15}N abundances measured in the sample and an untreated control, respectively. a_{CDDP} corresponds to the ^{15}N isotopic enrichment level of the cisplatin applied in the treatment experiments, which was 98%. The derivation of N_{CDDP}/N_{tot} as a function of the ^{15}N abundances is described below.

According to the error propagation theorem, the standard deviation of (N_{CDDP}/N_{tot}) is related to the standard deviations of the measured abundances by

$$\sigma_{N_{CDDP}/N_{tot}} = \sqrt{\left(\frac{\partial \frac{N_{CDDP}}{N_{tot}}}{\partial a_{15N,tot}}\right)^2 \sigma^2_{a_{15N,\,tot}} + \left(\frac{\partial \frac{N_{CDDP}}{N_{tot}}}{\partial a_{15N,ctr}}\right)^2 \sigma^2_{a_{15N,\,ctr}}} \text{.* MERGEFORMAT (1.9)}$$

It should be noted that the term related to the ligands had to be excluded from Eq. * MERGEFORMAT (1.9) since the uncertainty in the isotopic enrichment level of the applied CDDP was not specified. After calculation of the partial derivatives, Eq. * MERGEFORMAT (1.9) becomes

$$\sigma_{N_{CDDP}/N_{tot}} = \frac{1}{\left(a_{15N,CDDP} - a_{15N,ctr}\right)^2}\sqrt{\left(a_{15N,tot} - a_{15N,ctr}\right)^2 \sigma^2_{a_{15N,ctr}} + \left(a_{15N,CDDP} - a_{15N,ctr}\right)^2 \sigma^2_{a_{15N,tot}}} \text{,* MERGEFOR}$$

For estimation of the analytical precision, the counting error (1σ) associated with the determination of the isotopic abundances (see Eq. * MERGEFORMAT (1.5) above) was inserted in Eq. * MERGEFORMAT (1.10).

From a more general perspective, the derivation of the algebraic function describing the relationship between the abundance of the isotopic label (here: a_{15N}) and the relative number of atoms originating from drug accumulation (here: $N_{CDDP}/N_{tot.}$) relies on the law of mass conservation, from which follows, that:

(i) For any considered element *X*, the total number of atoms contained in the analyzed region of a compartment is equal to the sum of atoms originating from the compounds in the compartment (referred to by measurement of an untreated control) and the number of atoms added through drug accumulation

$$n_{X,tot} = n_{X,ctr} + n_{X,drug}, \qquad \text{* MERGEFORMAT (1.11)}$$

(ii) The number of label atoms $n_{^AX}$ contained in the considered region of the compartment is equal to the sum of label atoms originating from the compounds in the compartment and the number of label atoms added through drug accumulation

$$n_{^AX,tot} = n_{^AX,ctr} + n_{^AX,drug}, \qquad \text{* MERGEFORMAT (1.12)}$$

Utilizing the isotopic abundance of the label atoms $a_{^AX}$, which is defined by

$$a_{^AX} = \frac{n_{^AX}}{n_{^AX} + n_{^BX} + n_{^CX}\ldots}, \qquad \text{* MERGEFORMAT (1.13)}$$

where the superscripts at *X (A, B, C…)* refer to the mass number of the respective isotopes, Eq. * MERGEFORMAT (1.13) can also be written as

$$n_{X,tot}a_{^AX,tot} = n_{X,ctr}a_{^AX,ctr} + n_{X,drug}a_{^AX,drug}. \qquad \text{* MERGEFORMAT (1.14)}$$

Rearrangement of this equation and utilizing the relationship $n_{X,ctr.} = n_{X,tot.} - n_{X,drug}$ (see Eq. * MERGEFORMAT (1.11)), an equation can be formulated that describes the fraction of drug atoms contained in the analyzed region

$$\frac{n_{X,drug}}{n_{X,tot.}} = \frac{a_{^AX,tot} - a_{^AX,ctr}}{a_{^AX,drug} - a_{^AX,ctr}}, \qquad \text{* MERGEFORMAT (1.15)}$$

which is actually the relationship that we were looking for.

Pt_{CDDP} / $N_{tot.}$. As already mentioned in the main text, SIMS is only semi-quantitative in elemental analysis, which is mainly due to the fact that the product of the sputter yield and the ionization probability of sputtered particles can vary over several orders of magnitude. However, utilizing an appropriate secondary ion signal as a reference, a relationship can be established in which the normalised intensity of the secondary ion signal associated with the analyte is linearly related to the concentration of the analyte. In the measurements on the resin section we selected the $^{12}C_2^-$ signal as a reference since it showed the lowest variability over the distinct cellular compartments (in comparison to $^{12}C^-$ and $^{16}O^-$, which can also be considered as candidates for reference signals due to high concentration of carbon and oxygen in the resin, which represents the matrix in such samples). As such, the $^{194}Pt^-/^{12}C_2^-$ and $^{12}CN^-/^{12}C_2^-$ signal intensity ratios served as indicators of the local platinum and nitrogen contents. The designation $^{12}CN^-$ for the cyanide ions has deliberately been chosen to indicate that the relative ion yields of $^{12}C^{14}N^-$ and $^{12}C^{15}N^-$ also depends on the nitrogen isotopic composition. In other words, any constriction to the $^{12}C^{14}N^-$ signal leads to an underestimation of the local nitrogen content since the contribution of the highly ^{15}N enriched ammine ligands is neglected. This has to be particularly noted when the two isotopic ions cannot be detected simultaneously, which is actually the case in elemental mapping of high mass number elements, such as gold or platinum. Consequently, we first calculated the averages of the $^{12}C^{14}N^-/^{12}C_2^-$ intensity ratios determined in the platinum and nitrogen isotope measurements,

$$\left(\frac{12C14N^-}{12C_2^-}\right)_{avg} = \frac{1}{2}\left[\left(\frac{12C14N^-}{12C_2^-}\right)_{Pt} + \left(\frac{12C14N^-}{12C_2^-}\right)_{15N}\right],\text{* MERGEFORMAT (1.16)}$$

from which the total $^{12}CN^-$ signal intensity was calculated by consideration of the contribution of ^{15}N containing ions, which was evaluated in the nitrogen isotope measurements, i.e.

$$\left(\frac{12CN^-}{12C_2^-}\right)_{avg} = \left(\frac{12C14N^-}{12C_2^-}\right)_{avg} \times \left(1 + R_{15N/14N}\right).\text{* MERGEFORMAT (1.17)}$$

The local relative accumulation of platinum originating from CDDP uptake was referred to by subtraction of ROI specific $^{194}Pt^-/^{12}C_2^-$ intensity ratios measured on an untreated control *('ctr')* from the values determined on the respective samples (designated, for the sake of consistency, with the ligands, as *'total'*),

$$\left(\frac{194Pt^-}{12C_2^-}\right)_{CDDP} = \left(\frac{194Pt^-}{12C_2^-}\right)_{tot} - \left(\frac{194Pt^-}{12C_2^-}\right)_{ctr}.\text{* MERGEFORMAT (1.18)}$$

Finally, the platinum signal intensities were normalised to the $^{12}CN^-$ signal intensities,

$$\frac{194Pt^-_{CDDP}}{12CN^-} = \left(\frac{194Pt^-}{12C_2^-}\right)_{CDDP} \times \left(\frac{12CN^-}{C_2^-}\right)_{avg}^{-1},\text{* MERGEFORMAT (1.19)}$$

which, by definition, is proportional to the concentration ratio of the platinum originating from CDDP to the total nitrogen content in the considered compartments,

$$\frac{Pt_{CDDP}}{N_{tot}} \propto \frac{194Pt^-_{CDDP}}{12CN^-}$$, presented in arbitrary units [a.u.].* MERGEFORMAT (1.20)

According to the error propagation theorem, the standard deviation of the signal intensity ratio is given by

$$\sigma_{194Pt^-_{CDDP}/12CN^-} = \left(\frac{12CN^-}{12C_2^-}\right)^{-1}_{avg} \sqrt{\sigma^2_{\left(194Pt^-_{CDDP}/12C_2^-\right)_{Pt}} + \left(\frac{194Pt^-_{CDDP}}{12C_2^-}\right)^2_{Pt} \sigma^2_{\left(12CN^-/12C_2^-\right)_{avg}}}$$.* MERGEFORMAT

For estimation of the analytical precision, the variances can be calculated from the measured signal intensities (in counts) as

$$\sigma^2_{194Pt^-_{CDDP}/12C_2^-} = \frac{1}{4}\left[\left(\frac{194Pt^-}{\left(12C_2^-\right)^2} + \frac{\left(194Pt^-\right)^2}{\left(12C_2^-\right)^3}\right)_{ctr} + \left(\frac{194Pt^-}{\left(12C_2^-\right)^2} + \frac{\left(194Pt^-\right)^2}{\left(12C_2^-\right)^3}\right)_{tot}\right]$$ * MERGEFORMAT

$$\sigma^2_{12CN^-/12C_2^-} = \left(1 + R_{15N/14N}\right)^2 \sigma^2_{\left(12C14N^-/12C_2^-\right)_{avg}} + \left(\frac{12C14N^-}{12C_2^-}\right)^2_{avg} \sigma^2_{R_{15N/14N}}$$,* MERGEFORMAT (1.2

where

$$\sigma^2_{\left(12C14N^-/12C_2^-\right)_{avg.}} = \frac{1}{4}\left[\left(\frac{12C14N^-}{\left(12C_2^-\right)^2} + \frac{\left(12C14N^-\right)^2}{\left(12C_2^-\right)^3}\right)_{Pt} + \left(\frac{12C14N^-}{\left(12C_2^-\right)^2} + \frac{\left(12C14N^-\right)^2}{\left(12C_2^-\right)^3}\right)_{15N}\right]$$ * MERGEFC

and

$$\sigma^2_{R_{15N/14N}} = \frac{12C15N^-}{\left(12C14N^-\right)^2} + \frac{\left(12C15N^-\right)^2}{\left(12C14N^-\right)^3}$$ * MERGEFORMAT (1.25)

References

1. S. C. Dhara, *Indian. J. Chem.*, 1970, **8**, 193-194.
2. Y. Y. Scaffidi-Domianello, A. A. Legin, M. A. Jakupec, A. Roller, V. Y. Kukushkin, M. Galanski and B. K. Keppler, *Inorg. Chem.*, 2012, **51**, 7153-7163.
3. G. Slodzian, F. Hillion, F. J. Stadermann and E. Zinner, *Appl. Surf. Sci.*, 2004, **231-232**, 874-877.

4. G. Slodzian, M. Chaintreau, R. Dennebouy and A. Rousse, *Eur. Phys. J-Appl. Phys.*, 2001, **14**, 199-231.
